# Green extraction routes for conversion of *Nerium oleander* L. biomass into bioactive agents: phytochemical insights and antimicrobial promise

**DOI:** 10.1186/s12906-026-05594-x

**Published:** 2026-10-06

**Authors:** Mohamed T. Selim, Nashaat N. Mahmoud

**Affiliations:** Botany and Microbiology Department, Faculty of Science, Al-Azhar University, Nasr City, Cairo, 11884 Egypt

**Keywords:** *Nerium oleander* L., Phytochemical, Carbohydrates, Lipids, Proteins, Antimicrobial

## Abstract

**Background:**

*Nerium oleander *L. (*N. *oleander) is a species of shrub or small tree in the Apocynaceae family that has high levels of digitalin linear activity and cardiac glycosides, especially nerine and oleander. The study was researched to assess the *in vitro* proximate compositions, phytochemical analysis, and antimicrobial activity of the dried stems, leaves, and flowers of *N. oleander* extracted using various solvents.

**Methods:**

Petroleum ether, ethyl acetate, acetone, methanol, and water extracts of the various solvents were tested for total phenol, flavonoid, and tannin contents using various phytochemical assays, while the antimicrobial activity was evaluated using the agar well diffusion method against six bacterial and fungal strains.

**Results:**

The flowers had a higher moisture content (9.40%), whereas the stems had a higher ash content (18.58%) than the leaves and flowers. The flowers also had higher lipid, carbohydrate, and crude protein contents (4.33, 66.16, and 15.29%, respectively). The leaves of the *N. oleander* produced a maximum yield followed by flowers and stems, respectively. The highest percentage extractive yield was demonstrated by water extracts, which were followed by methanol, acetone, ethyl acetate petroleum ether extracts. The findings of the study showed that alkaloids, flavonoids, tannins, glycosides, steroids, coumarins, quinones, phenols, cardiac glycosides, and terpenoids were found by phytochemical analysis of *N. oleander* in different parts, while saponins and anthraquinones were completely absent in all parts. On the other hand, anthocyanins are present only in flowers and completely absent in stems and leaves. The greatest levels of phenol content, flavonoids and tannins were found in acetone extract of the flowers (157.12 ± 17.62 mg GAE/g, 187.43 ± 15.91 mg QE/g, and 89.93 ± 18.77 mg TAE/g, respectively), while the petroleum ether extract of stems had the lowest amount (5.45 ± 2.65 mg GAE/g, 6.43 ± 0.29 mg QE/g and 3.45 ± 2.05 mg TAE/g, respectively). Antimicrobial tests revealed the extract’s ability to inhibit several Gram-positive bacteria (*Staphylococcus aureus* ATCC 6538 and *Bacillus subtilis* ATCC 6633), Gram-negative bacteria (*Pseudomonas aeruginosa* ATCC 9027, *Salmonella Typhimurium* ATCC 14028 and *Escherichia coli* ATCC 11229), and eukaryotic strains such as unicellular fungi (*Candida albicans* ATCC 10231).

**Conclusion:**

These results highlight the potential of *N. oleander* extracts as natural antimicrobials.

**Graphical Abstract:**

**Figure:**
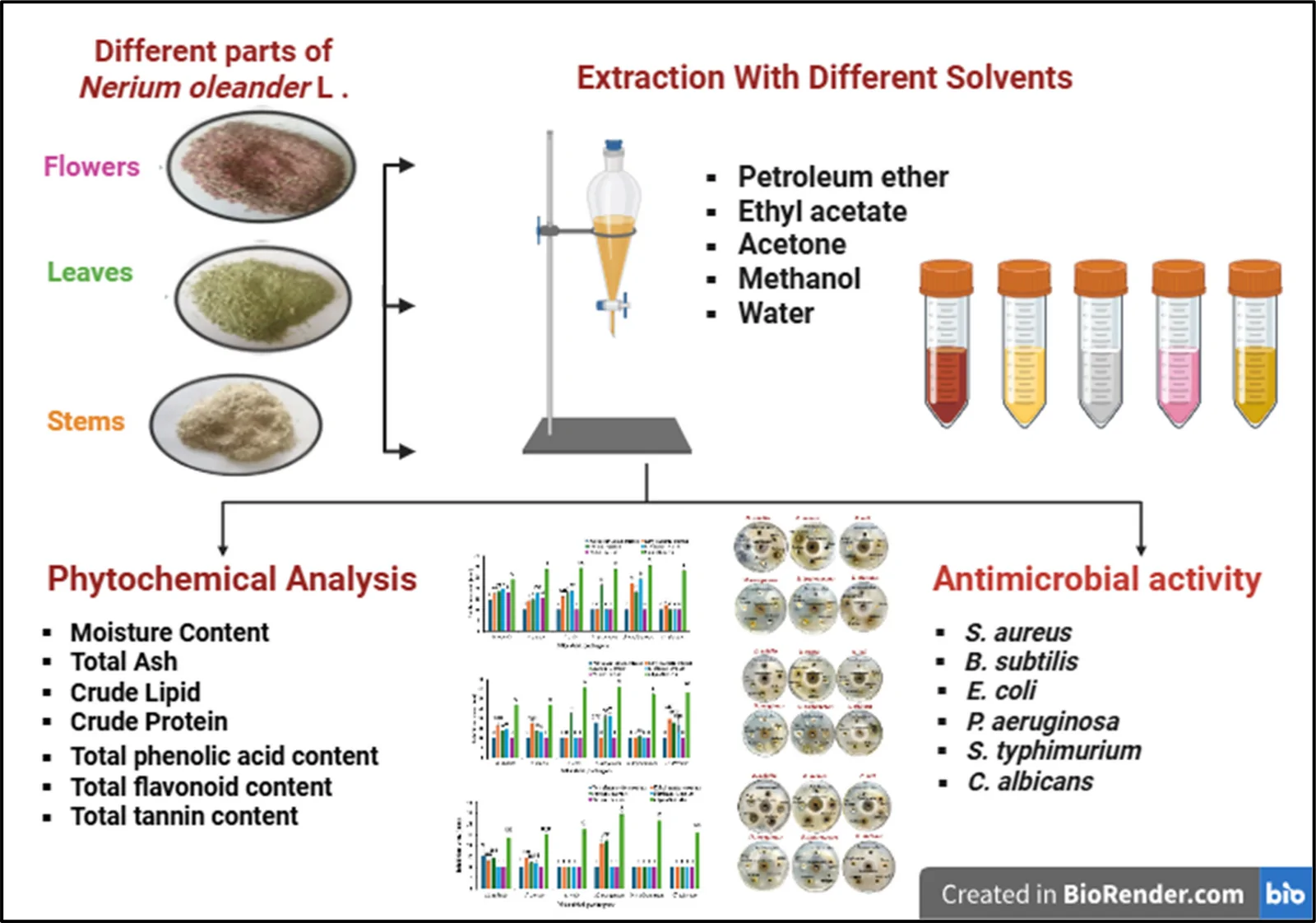

## Introduction

Plants are a significant source of many different secondary metabolites that are used to treat and prevent illnesses [1, 2]. By isolating and describing the active ingredients in many plants, their pharmacological and biological effects were elucidated [3]. These active ingredients are now also utilized as raw materials in pharmaceuticals [4, 5]. Many medicines are manufactured from compounds derived from herbal secondary metabolites [6–9]. Many properties, including antioxidant, antimalarial, antiviral, analgesic, antihelminthic, antibacterial, diuretic, anticancer, anti-inflammatory, antiallergic, antifungal, anti-urolithiasis and, antimutagenic, have been found in secondary metabolites that ensure plant survival [10–15].

Plants can yield a variety of phytochemicals that are highly advantageous to humans, and medicinal plants are now the most abundant biological source of these compounds. These chemicals are used to make both traditional and modern medications, food supplements, nutraceuticals, folk remedies, and pharmaceutical intermediates [16, 17].

A group of synthetic or biosynthetic chemical substances known as antimicrobial chemicals either kill or effectively inhibit the growth and metabolism of a wide range of microorganisms [18, 19]. In several countries, pharmacologically significant plants are used as drugs and provide a wealth of antibacterial compounds [20, 21].

Plant extracts are being used by researchers worldwide because of their antiviral, [16] antibacterial [15], and antifungal qualities [22]. The components of plant oils and extracts utilized in the pharmaceutical and other sectors are what give plants their antibacterial properties [23].

*Nerium oleander* L., a shrub or small tree that is typically found in warm and subtropical climates, is a member of the *Nerium* genus and the Apocynaceae family [24, 25]**.** Terminal clusters of varied-colored, five-petaled blooms, about 5 cm in diameter, are produced by this plant. Digitalin linear activity is high in *N. oleander* due to the presence of cardiac glycosides, specifically nerine and oleander. Many biological and pharmacological activities have been demonstrated by the abundant phytochemical content of *N. oleander*. There are extremely toxic and deadly glycosides and alkaloids in every part of the oleander plant [24]. They have different physiological effects: antibacterial [26], antimicrobials [27], antiulcer [28], hepatoprotective [29], anticarcinogenic [30, 31], cytotoxic, antidiarrheal [32], larvicides [33], and ani-anthelminthic activities [34].

The leaves of *N. oleander* have diaphoretic, diuretic, antibacterial, cardiotonic, and anticancer properties [35], and anti-fungal [36]. The bark has diuretic, emetic, diaphoretic, cardiac tonic, and expectorant properties [37], as well as steroids that have therapeutic qualities like anti-inflammatory, hepatoprotective, anti-cancer, antipyretic, antioxidant, antifungal, and anti-HIV effects [38], the active pharmacological components of *N. oleander* have also been identified as terpenes, flavonoids, and polyphenols [39]. Additionally, previous investigations have demonstrated the diaphoretic, emetic, diuretic, cardiotonic, sternutatory, and expectorant properties of *N. oleander* flowers [40].

This investigation aimed to assess the *in vitro* proximate composition, phytochemical profile, and antimicrobial potential of dried stems, leaves, and flowers of *N. oleander* extracted using solvents of varying polarities. To the best of our knowledge, this is the first comprehensive comparative study to examine the phytochemical and antimicrobial properties of three distinct plant parts (stems, leaves, and flowers) of *N. oleander* using five different solvents.

This study offers several novel contributions: a comparative analysis of stems, leaves, and flowers of *N. oleander* under consistent conditions; evaluation of extraction efficiency using solvents of varying polarities; broad-spectrum antimicrobial testing against bacteria and fungi; and correlation of phytochemical content with biological activity. This dual comparison across plant parts and solvents provides new insights for targeted extraction and pharmaceutical applications.

## Materials and methods

### Plant material

**Fig. 1:**
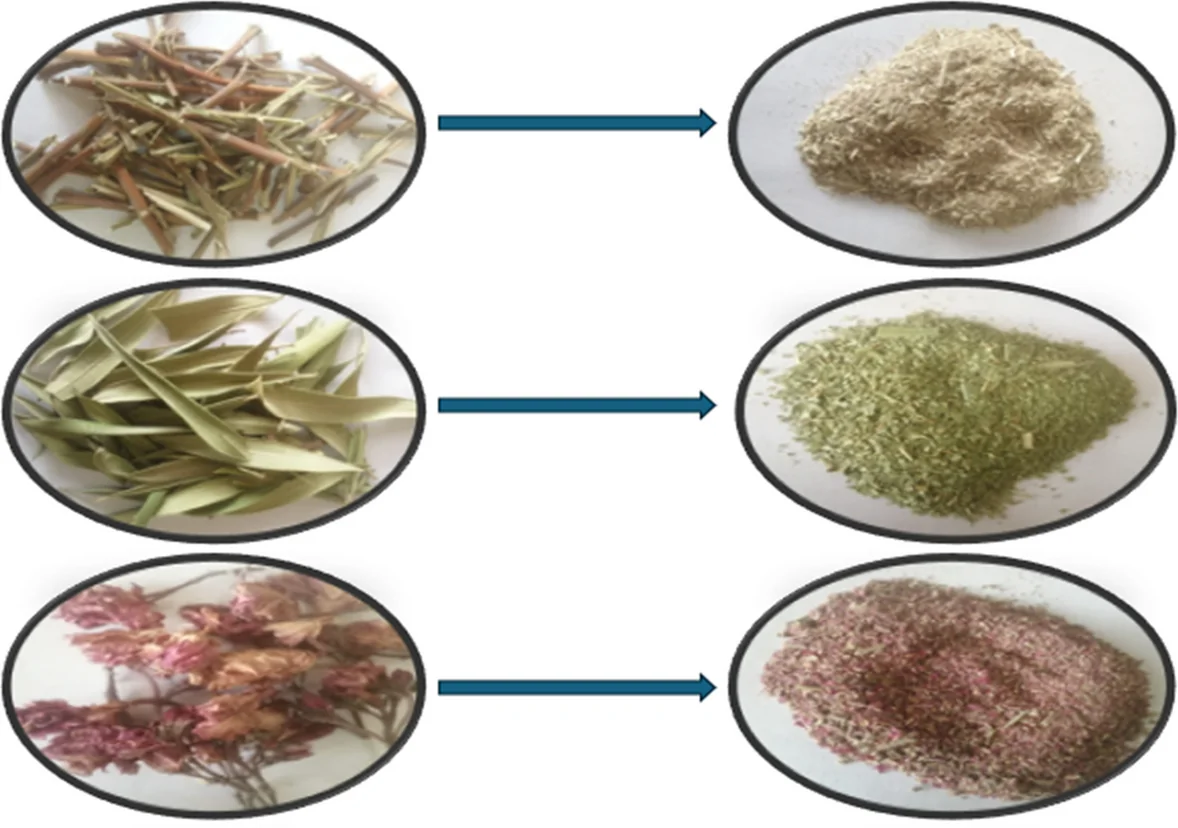
Appearance of the stem, leaf, and flower tissues of N. oleander both before and after grinding

Stem, leaf, and flower parts of *N. oleander* were collected from the Garden of the Faculty of Science, Al-Azhar University for Boys, Cairo, in April 2024. Prof. Dr. Osama Gamal El-Basouny, Assistant Professor of Plant Taxonomy, Botany and Microbiology Department, Faculty of Science, Al-Azhar University, Egypt, authenticated the plant sample. A voucher herbarium specimen (AZU/SCI/BOT/HERB/2024–028) was deposited in the Herbarium of the Botany and Microbiology Department, Faculty of Science, Al-Azhar University, Cairo, Egypt. To remove impurities, distilled water was used to wash the fresh parts. The homogenized, disease-free, and unblemished parts were then left to dry at laboratory temperature in the shade until their weight remained constant. They were then ground into a fine powder using an electric grinder (Fig. 1), sieved, and collected in glass vials [41].

### Preparation of extracts

Using a Soxhlet apparatus, ground stems, leaves, and flowers from *N. oleander* (100 g each) were extracted one by one using different organic solvents according to their polarity. This began with petroleum ether (non-polar), ethyl acetate (moderately polar), acetone (intermediate polarity), methanol (high polarity), and water (highly polar), at temperatures below the boiling point of the solvents. To investigate the phytochemical analysis and antimicrobial properties of the obtained samples, a rotary evaporator was used to collect the filtrate and evaporate any excess solvent at a temperature of no more than 45 °C while applying reduced pressure. The samples were then stored at 4 °C in the dark [41]. The obtained yield was measured by applying the following equation [42].

$$\mathrm{Yield}\left(\%\right)=\left[\left(\text{Weight of the extract}\right)/\left(\text{Weight of the dried sample}\right)\right]\times 100$$

### Determination of proximate composition

#### Moisture content

Moisture content was carried out by using the method described by Arunachalam and Parimelazhagan, [43]. Use the appropriate drying equipment to dry the sample to a constant weight at a temperature of no more than 115 °C after immediately weighing 10 g of fresh material and recording it as "weight of fresh sample." Let the sample cool. After the sample has cooled, weigh it once more and note its "weight of dry sample." Using the following formula, the moisture content is determined:

$$\begin{aligned}\mathrm{Moisture}\left(\%\right)=&[(\text{Weight of fresh sample-Weight of dry sample})\\ &/(\text{Weight of the fresh sample})]\times 100\end{aligned}$$

#### Total ash

Five grams of dried powder were burnt to 600 ˚C in a furnace for three hours, and then the mixture was cooled. Concerning the air-dried orange peel sample, the ash was weighed, and the percentage was measured [44].

$$\mathrm{Ash}\left(\%\right)=\left[\left(\text{Weight of sample after burning}\right)/\left(\text{Weight of sample before burning}\right)\right]\times 100$$

#### Crude lipid

The crude lipid content of different parts of *N. oleander* was estimated by Soxhlet method [45] with slight modification. A 10 g of dry sample was extracted with 250 mL of petroleum ether (60–80 °C) for 6 h. The extract was evaporated to dryness at 102 °C to a constant weight, then cooled in a desiccator and weighed. The fat content was calculated based on the weight difference and expressed as a percentage of the dry weight.

$$\begin{aligned}\% \text{Crude lipid}&=[(\text{Weight of flask and extracteds lipid}\\ &-\text{Weight of empty flask})/(\text{Weight of sample taken})]\times 100\end{aligned}$$

#### Crude protein

The crude protein content of different parts of *N. oleander* was estimated by Krishna et al., [46]. 

A 100 mg sample was homogenized in 10 mL of 0.2 M phosphate buffer, filtered, and centrifuged at 3000 × g for 10 min. The upper liquid was diluted to 10 mL with distilled water. A 1 mL of the diluted extract was reacted with 5 mL of alkaline copper reagent and incubated for 10 min at room temperature. Then, 0.5 mL of Folin–Ciocalteau (1 N) reagent was added. After 30 min in the dark, the absorbance was measured at 660 nm. The protein concentration was calculated using a standard curve for bovine serum albumin (BSA) and expressed as BSA equivalents in mg per 100 g of dry sample.

The difference [100 − (moisture content + crude protein + crude lipid + total ash)] indicates the amount of carbohydrates. The nutritional value was determined using the technique outlined by Indrayan et al. [47].

### Preliminary phytochemical screening

Using the techniques outlined by Harborne, [48] a qualitative phytochemical analysis of the plant extracts was performed, to look for the presence of phenols, flavonoids, alkaloids, quinones, steroids, terpenoids, glycosides, saponins, anthraquinone, tannins, anthocyanins, cardiac glycosides, and coumarins.

### Quantification of total phenolic content

Using the Folin–Ciocalteau method, which was characterized by Chandra et al. [49], the total phenolics in different parts of *N. oleander* were quantified. Briefly, 50 µL of each extract (1 mg/mL) or standard (0–50 µg/mL) was mixed with 0.2 mL of 0.5 M Folin–Ciocalteu reagent and 0.6 mL distilled water. After 15 min of shaking, 1.0 mL of 8% Na₂CO₃ was added, and the volume was adjusted to 3.0 mL. The mixture was incubated in the dark for 30 min, and absorbance was measured at 760 nm. The assay was performed in triplicate. Total phenolic content was determined using the standard curve (y = 0.003x + 0.0237; R^2^ = 0.9976) and calculated using the formula CV/m, expressed as mg gallic acid equivalent per gram (mg GAE/g) of extract.

### Quantification of total flavonoid content

Using a slight modification aluminum chloride calorimetric method first described by Aryal et al. [50], we measured the total flavonoid content in different parts of *N. oleander*. The amount of yellowish-orange coloration produced by the flavonoid-aluminum chloride complex reaction is a key variable in this process. Briefly, 2 mL of distilled water, 0.15 mL of 5% w/v NaNO_2_, and 500 μL of plant extract (1 mg/mL) were combined. The incubation period was 5 min. After waiting another 5 min, a solution was made by combining 0.15 mL of 10% w/v AlCl_3_ with 1 mL of NaOH (4%,w/v). The next step was to vortex the mixture and then incubate it at 40 °C for 15 min. Using the same method, we also made a quercetin standard solution of variable concentrations (0–200 μg/mL). The study was replicated three times. We used the estimated equation to determine the flavonoid content, y = 0.0034_X_ + 0.0135, with an R^2^ value of 0.9971. After calculation determine the flavonoid content of the sample and report the result in mg quercetin equivalents/g (mg QE/g).

### Quantification of total tannin content

The total tannins were assessed using the Folin-Denis spectrophotometer technique created by Makkar [51]. and Mahmoud et al. [15]. A 500 μL of each extract (1 mg/mL) was mixed with 100 mg of polyvinyl polypyrrolidone and diluted with 0.5 ml of distilled water. The tubes should be incubated for 4 h at 4 °C. After centrifuging for 10 min at 4 °C at 3000 rpm, only non-tannin phenolics are found in the upper liquid. Take 100 μL of non-tannin phenolics and 0.5 mL of the Folin-Ciocalteu reagent (1 N) in triplicates. Then incubate for 5 min. Including the blank, add 2.5 mL of Na_2_CO_3_ (5%, w/v). Mix and incubate for 40 min in the dark. At 725 nm, absorbance was determined with a UV spectrophotometer. By utilizing the calibration graph equation (y = 0.0086x + 0.0233, R^2^ = 0.9926) and the CV/m formula, tannin was determined and expressed as mg TAE/g.

### Antimicrobial activity

To explore the antimicrobial potential of different parts of *N. oleander* extracts (stems, leaves, and flowers) that were extracted using different solvents, various pathogenic strains were tested. These included Gram-positive bacteria (*S. aureus* ATCC 6538 and *B. subtilis* ATCC 6633), Gram-negative bacteria (*P. aeruginosa* ATCC 9027, *S. typhimurium* ATCC 14028 and *E. coli* ATCC 11229), and eukaryotic strains such as unicellular fungi (*C.albicans* ATCC 10231). The agar well diffusion method was employed to test the efficacy [52]. For 48 h at 37 °C, all of the bacteria used in this study were subcultured in nutrient agar (NA). Before the cultures were inoculated onto 15 cm-diameter NA dishes, they were set to a McFarland turbidity standard of 0.5. To achieve the required concentrations, DMSO was independently diluted in each sample. Six wells, each 0.6 mm in diameter, were made on each agar plate using a sterile cork borer. Then, 100 µL of each extract was added to the wells. Additionally, ciprofloxacin was also added in this experiment as the positive control. After an hour of refrigeration, the plates were incubated at 35 ± 2 ◦C for 24 h. The diameter of the clear zone surrounding each well was measured in millimeters. The experiment was conducted in triplicate.

### Statistical analysis

Data were analyzed using analysis of variance (ANOVA), and Tukey's HSD test was used to determine whether the mean difference between treatments was statistically significant at the *p* < 0.05 level. As in our previous study, Minitab® version 18 (2017) was used to perform the statistical analysis [53, 54].

## Results and discussion

### Extractive yield and color of extracts

The various components were recovered by utilizing the color of the *N. oleander* extracts made with petroleum ether, ethyl acetate, acetone, methanol, and water extract. As shown in Table 1, the various parts display a range of extract colors, including green, light green, yellow-green, brown-green, brown, yellow–brown, and dark brown. The results aligned with previous findings in *Centaurea calcitrapa* L., where the aerial flowering parts displayed different extract colors, such as yellow–brown, green, dark green, and dark brown, when dissolved in chloroform, ethyl acetate, methanol, and water, respectively [41].

**Table 1:** The percentage yield and color of fractionated plant extracts

Parts Solvent used	Boiling Point	Total hrs. of extraction	% Yield	Color of extract
Petroleum ether	60 ^◦^C	5 h	8.27 ± 0.42^i^	Green
Ethyl acetate	68 ^◦^C	6 h	8.58 ± 0.29^i^	Light green
Stems Acetone	56 ^◦^C	6 h	12.17 ± 0.38^gh^	Yellow–brown
Methanol	80 ^◦^C	8 h	18.20 ± 0.72^e^	Brown
Water	95 ^◦^C	10 h	36.50 ± 0.91^b^	Dark brown
Petroleum ether	60 ^◦^C	5 h	12.27 ± 0.31^gh^	Green
Ethyl acetate	68 ^◦^C	6 h	15.75 ± 0.5^f^	Green
Leaves Acetone	56 ^◦^C	6 h	18.77 ± 0.68^de^	Brown-green
Methanol	80 ^◦^C	8 h	22.17 ± 0.38^c^	Brown- green
Water	95 ^◦^C	10 h	40.22 ± 1.05^a^	Dark brown
Petroleum ether	60 ^◦^C	5 h	11.30 ± 0.72^ h^	Yellow-green
Ethyl acetate	68 ^◦^C	6 h	9.25 ± 0.5^i^	Yellow-green
Flowers Acetone	56 ^◦^C	6 h	13.42 ± 0.29^ g^	Brown
Methanol	80 ^◦^C	8 h	20.33 ± 1.04^ cd^	Brown
Water	95 ^◦^C	10 h	39.22 ± 1.01^a^	Dark brown
Statics S.S	42.04			
M.S	5.26			
Df	8			
*F*	11.7			
*P*-value	0.001			

Data in columns are shown as mean values ± standard deviation with different superscripts are significantly different according to Tukey's "HSD" (*p* < 0.05). Mean square values ​​(M.S); the sum of squares values ​​(S.S); significance (*f*); degrees of freedom (Df). Superscripts (a, b, c, d, e, f, g, h, i) (indicate pairwise comparisons if they are statistically different)

The type and effectiveness of the solvent have a direct impact on the extraction yield. Production expenses like solvent volume, energy costs, and extraction time, as well as the effects on the environment and people, are all related to extraction efficiency [55]. Therefore, choosing an appropriate solvent that is economical and environmentally friendly is crucial for extracting bioactive phytochemicals from plant materials. In the present study, three distinct parts of *N. oleander* (stem, leaf, and flower) were selected, and solvents with varying polarities (petroleum ether, ethyl acetate, acetone, methanol, and water) were used to isolate the bioactive compounds from the dried plant material.

The resultant yield extracted from different parts of *N. oleander* by different solvents was calculated and presented also in Table 1. The results indicated that *N. oleander* leaves produced the highest yield, followed by flowers and stems, respectively. Among the extraction solvents, aqueous extracts showed the highest extraction yield, followed by methanol, acetone, ethyl acetate, and petroleum ether extracts. Comparable trends in extractive yield have been reported previously: Saeed and Shabbir [56] observed that methanol (11.5%) extracted more from the whole *Torilis leptophylla* plant than chloroform (4.3%) or hexane (5.4%), while Ammar et al. [57] found that methanol (14.82%) was also the most effective solvent for the flowers of *Opuntia ficus-indica*, compared with hexane (2.86%). Likewise, Zaid et al. [58] found that after 48 h of extraction using a 1:1 (v/v) methanol: distilled water solution, the percentage extractive yield of *N. oleander* leaves was 36%.

This is explained by the polar protic nature of water and methanol and their higher dielectric constants compared to acetone, ethyl acetate, and petroleum ether. This allows polar secondary metabolites and some non-polar secondary metabolites to be dissolved more readily in the former than in the latter [59]. The polarity of specific compounds found in plant parts is thought to be responsible for the variance in extractable matter yield in different solvents. This suggests that both internal and external influences may have an impact on the origin of the bioactive principle of medicinal plants [60]. High extractive yields in water and alcohol indicate the presence of polar compounds, including glycosides, tannins, and phenols, as widely reported in studies on plant secondary metabolites [61–63].

In plants, biologically active substances are typically found in trace amounts. An extraction method is one that can produce high-yield extracts with little alteration to the necessary functional characteristics of the extract [64]. The biological activities of extracts made with various extraction methods have been found to vary in a number of studies. Thus, the properties of the sample matrix, the chemical characteristics of the analytes, the matrix-analyte interaction, efficiency, and the desired properties must all be taken into consideration when choosing the appropriate extraction technique and solvent [60, 65].

### Proximate composition

The proximate composition of *N. oleander* stems, leaves, and flowers is presented in Table 2. Moisture content was highest (9.40%) in the flowers and lowest (7.97%) in the stems. Due to the relatively low moisture content, stems, leaves, and flowers are expected to have excellent storage quality, firm texture, and a longer shelf life. Low moisture content helps reduce the risk of microbial growth, unwanted fermentation, premature seed germination, and many other undesirable biochemical changes typically associated with high humidity levels [66].

**Table 2:** Proximate chemical composition of dried stems, leaves, and flowers of *N. oleander*

Components	Stems	Leaves	Flowers	*P*-value
Moisture (%)	7.97 ± 0.21^a^	8.07 ± 0.12^a^	9.40 ± 1.91^a^	0.285
Lipid (%)	2.90 ± 0.53^a^	3.20 ± 0.35^a^	4.33 ± 1.15^a^	0.127
Ash (%)	18.58 ± 0.63^a^	14.55 ± 1.58^b^	4.83 ± 1.04^c^	0.001
Protein (%)	8.69 ± 0.46^b^	10.41 ± 1.54^b^	15.29 ± 1.47^a^	0.002
Carbohydrate (%)	61.86 ± 2.36^a^	63.78 ± 2.2^a^	66.16 ± 4.15^a^	0.294
Nutritive value (Kcal/100 g)	308.31 ± 3.05^b^	325.56 ± 6.45^b^	364.79 ± 12.58^a^	0.001

The results (*n* = 3) are presented as mean ± standard deviation. A significant difference (*P* < 0.05) is indicated by different letters on the same row. If pairwise comparisons are statistically different, they are indicated by the superscripts a, b, and c

Flowers show the highest lipid content (4.33%) followed by leaves (3.20%) and the lowest in stems (2.90%). The lipid content of *N. oleander* was higher than that of the leaves of *Fagonia cretica*, *Pisum sativum,* and *Brassica oleracea* [67], and like that of wheat (2.83 g 100g^−1^ DW) [68]. The differences are likely related to factors such as plant growth stages, climate, and geography.

Ash content reflects the quantity of mineral elements present in plants [69]. Ash value is one of the common characteristics among the numerous physicochemical characteristics analyzed in this study that is used to identify and determine the purity of the plant material, especially when it is in powder form for use in a future study or application. The ash values reveal the presence of a variety of impurities, including silicate, oxalate, phosphate, and carbonate. These impurities might originate from plants (natural or physiological ash) or external materials, including sand and soil that sticks to plant surfaces (non-physiological ash) [70]. It is worth mentioning that the stems gave the highest significant increase in ash content (18.58%) compared to leaves and flowers (14.55% and 4.83%), respectively. This may be due to the increase of total ion accumulation because of increasing soil moisture stress and soil salinity, which agreed with the results obtained by Larcher, [71]. High ash content is useful for evaluating the quality of plant classification and provides an indication of the mineral content present in the sample [72].

The crude protein level was significantly (*p* < 0.05) increased in flowers (15.29%) compared to leaves and stems (10.41% and 8.69%), respectively. Sufficient protein concentration in plants can facilitate the synthesis of hormones that regulate several physiological processes, including growth, tissue repair, and maintenance of body protein [73]. It also indicates the potential benefit of *S. marianum* because proteins are essential for the synthesis of body tissues and regulated substances such as hormones and enzymes [74].

Carbohydrates are compounds produced during photosynthesis. A large amount of carbohydrates was observed in different parts of the plant under study. There was no significant difference between the stems, leaves, and flowers of *N. oleander* in the total carbohydrates (*P* < 0.05). The flowers show higher carbohydrate content (66.16%) followed by leaves (63.78%) and lowest in stems (61.86%). This is due to increasing soil moisture in the studied habitat increases the accumulation of carbohydrates remarkably, these results agree with those obtained by Escudero et al., [75]. The nutritional value was calculated by multiplying the protein, lipid, and carbohydrate values ​​by 4.00, 9.00, and 4.00, respectively, and then adding the results. The nutritional value was significantly (*p* < 0.05) increased in flowers (364.79 kcal/100 g) compared to leaves and stems (325.56 kcal/100 g and 308.31 kcal/100 g), respectively.

### Preliminary phytochemical screening

Phytochemicals continue to be used in both conventional and contemporary medical systems and are essential for the treatment of various illnesses. The qualitative chemical tests provide a variety of insights into the types of phytochemical components found in the crude medication. A qualitative analysis of the phytochemicals showed that different dried parts of *N. oleander* contained different phytoconstituents.

Table 3 presents an overview of the findings from the qualitative phytochemical screening process conducted in different parts to identify the secondary metabolites of *N. oleander.* Alkaloids, flavonoids, tannins, glycosides, steroids, coumarins, quinones, phenols, cardiac glycosides, and terpenoids were found by phytochemical analysis of *N. oleander* in different parts, while saponins and anthraquinones were completely absent in all parts. On the other hand, anthocyanins are present only in flowers and completely absent in stems and leaves. The findings of the phytochemical screening conducted in this work on different parts of *N. oleander* are consistent with Redha et al., [76].

**Table 3:** Preliminary phytochemical analysis of the dried stems, leaves, and flowers of *N. oleander*

Tested Compounds	Stems	Leaves	Flowers
Flavonoids	+	+	+
Tannins	+	+	+
Alkaloids	+	+	+
Steroids	+	+	+
Saponins	-	-	-
Phenols	+	+	+
Anthraquinones	-	-	-
Glycosides	+	+	+
Terpenoids	+	+	+
Quinones	+	+	+
Cardiac glycosides	+	+	+
Anthocyanins	-	-	+
Coumarins	+	+	+

+ (presence of phytoconstituents)

- (absence of phytoconstituents)

Polyphenols found in plants are a type of active ingredient that has antioxidant properties. In addition to their roles in preventing cancer and cardiovascular disease, they also have anticancer and antitumor effects [77]. The cosmetics, food, and medical industries all make extensive use of them [78]. The anti-aging and cell-degeneration properties of flavonoids are matched by their anti-cancer and anti-cholesterol properties, in addition to their capacity to regulate blood pressure and cholesterol levels [79]. Tannin is useful as an antidote for heavy metal and alkaloid poisoning, in addition to its antiviral and antibacterial properties. It can neutralize the body's superoxide free radicals and put off the aging process thanks to its powerful reduced properties [80].

### Total phenolic content (TPC)

**Fig. 2:**
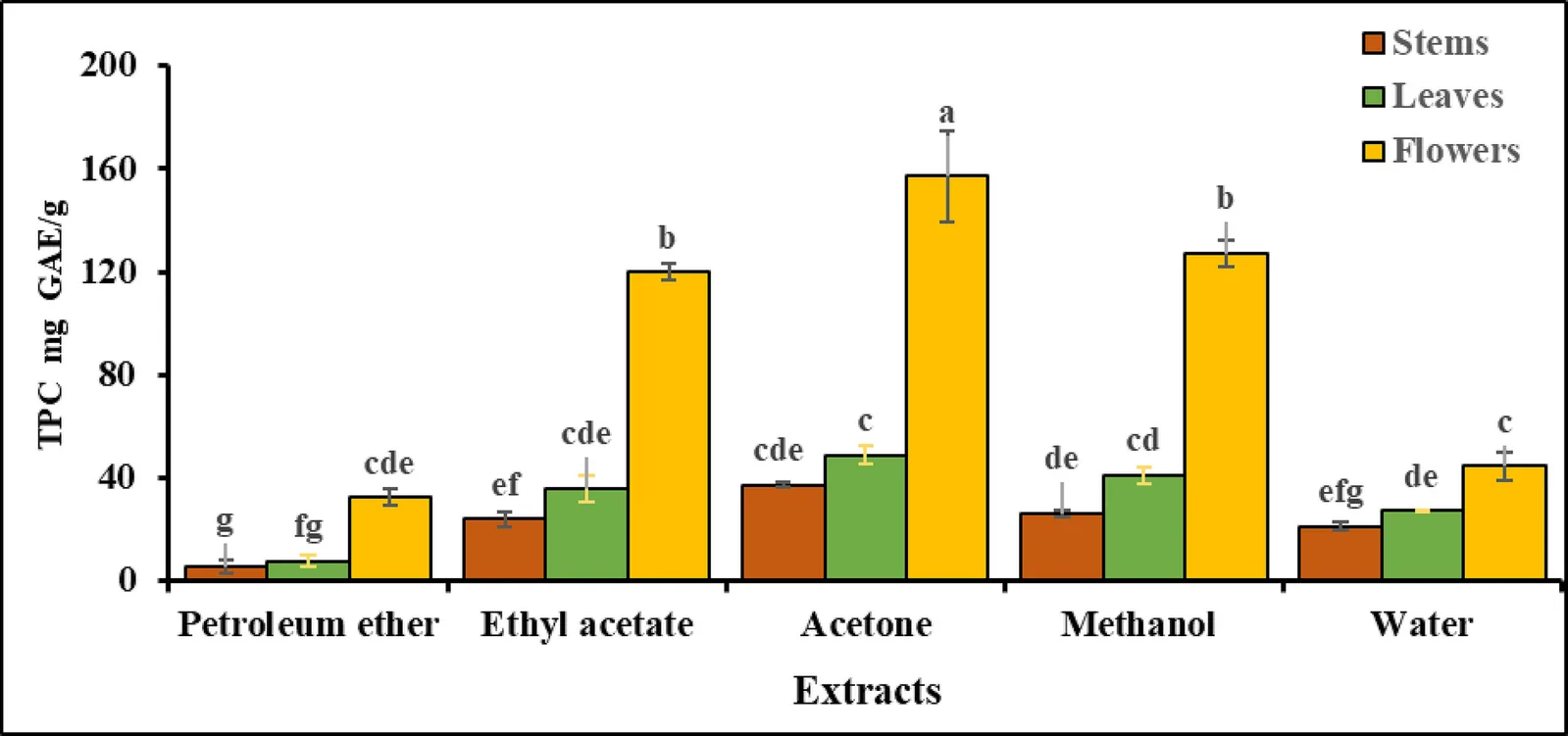
Total phenolic content (TPC) is expressed as mg gallic acid equivalent (GAE)/g of extract from different parts of N. oleander, using different solvent fractions in the standard equivalents. Values ​​are the mean ± standard deviation of three replicates. Superscripts: a, b, c, d, e, f, g (indicate pairwise comparisons if statistically different)

The results obtained showed that the content of phenolic varied from one part to another (flowers > leaves > stems) and from one extract to another (acetone > methanol > ethyl acetate > water > petroleum ether). The TPC of different extracts varied as a result of the solvent used and its polarity, and this was reported to play a major role in increasing phenol solubility (Fig. 2). The results of the TPC were expressed in mg of gallic acid equivalents per gram of extract (mg GAE/g). Acetone extract contains the highest percentage of phenols in the flowers (157.12 ± 17.62 mg GAE/g), While the petroleum ether extract of the stems contained the least amount (5.45 ± 2.65 mg GAE/g) (Table 4). The methanol and acetone extracts of flowers did not differ significantly (*P* < 0.05).

**Table 4:** A table summarizing the highest and lowest values for each secondary metabolite, the plant parts of *N. oleander* associated with these values, and the solvents that provided the most and least efficient extractions

Secondary metabolites	Values	Solvent	Part
Total phenolic content			
Highest value	157.12 ± 17.62	Acetone	Flower
Lowest value	5.45 ± 2.65	Petroleum ether	Stem
Total flavonoid content			
Highest value	187.43 ± 15.91	Acetone	Flower
Lowest value	6.43 ± 0.29	Petroleum ether	Stem
Total tannin content			
Highest value	89.93 ± 18.77	Acetone	Flower
Lowest value	3.45 ± 2.05	Petroleum ether	Stem

### Total flavonoid content (TFC)

**Fig. 3:**
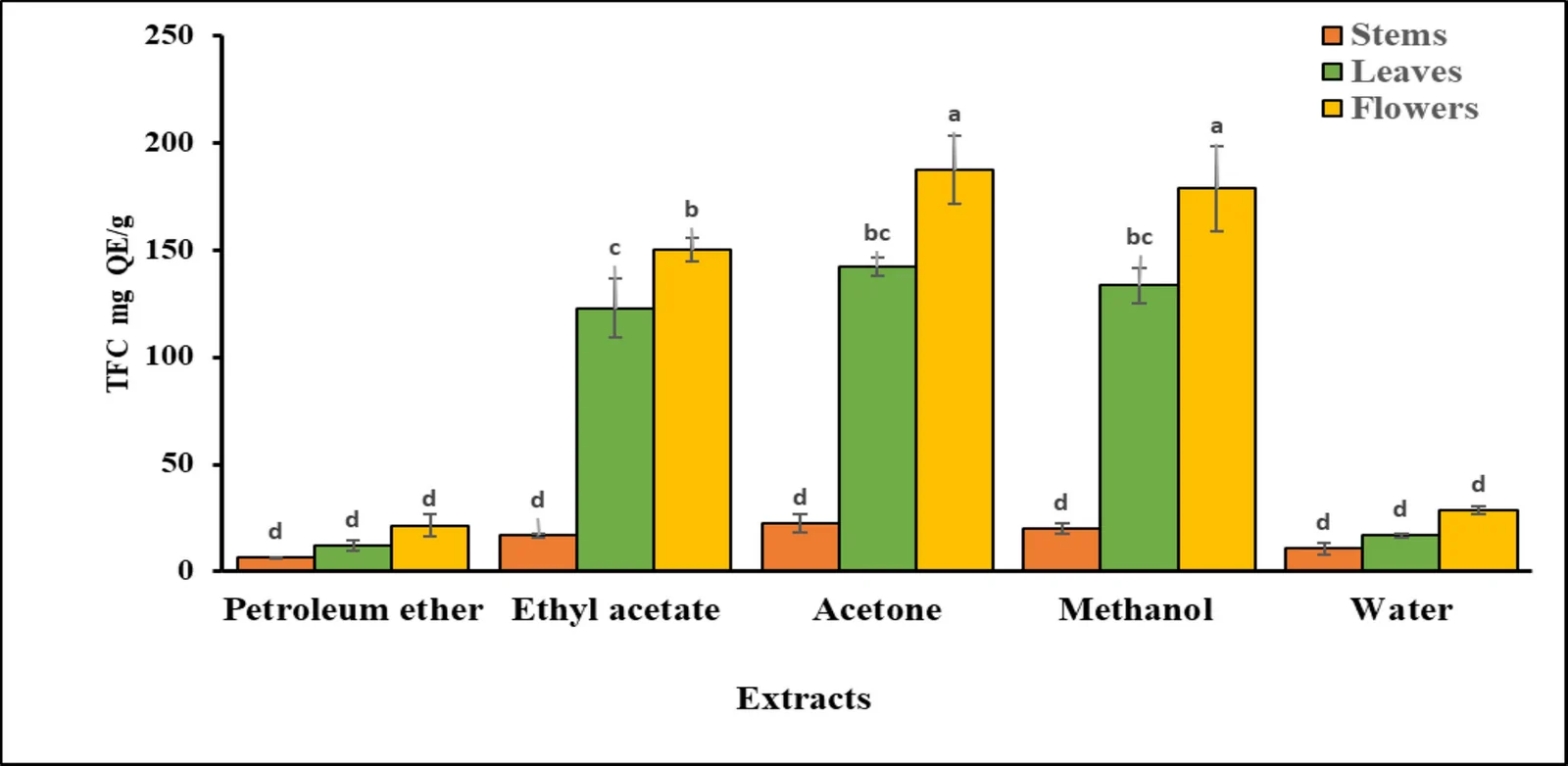
Total flavonoid content (TFC) is expressed in mg QE (quercetin equivalents)/g of extract from different parts of N. oleander, using different solvent fractions in the standard equivalents. Values ​​are the mean ± standard deviation of three replicates. Superscripts a, b, c, d (please indicate pairwise comparisons to see if they are statistically different)

Quercetin equivalent (mg QE/g) was used to express the TFC, which was calculated using standard quercetin as a reference. TFC was found to be higher in the acetone extract (Fig. 3), with the highest flavonoid content in the flowers compared to the leaves and stems. The evaluation result also revealed that the petroleum ether extract of stems had the lowest value of 6.43 ± 0.29 mg QE/g, while the acetone content in flowers was significantly higher (187.43 ± 15.91 mg QE/g) than the other solvent fractions (*P* < 0.05) (Table 4). No significant difference was observed between the methanol and acetone extracts of flowers and leaves (*P* < 0.05), nor between the percentages of acetone, methanol, and ethyl acetate in the stems (*P* < 0.05). Additionally, no significant difference was observed between all parts when extracted with petroleum ether and water (*P* < 0.05). The impact of different solvents on flavonoid content was similar to that on phenolic content. Several studies have reported varying levels of flavonoid content in *N. oleander* [81].

Flavonoids and other phenolic compounds are commonly recognized as plant secondary metabolites, characterized by an aromatic ring structure that contains at least one hydroxyl group. Over 8,000 phenolic compounds have been identified as natural substances derived from plants [82, 83]. It is very interesting to note that half of these phenolic compounds are flavonoids that appear in the form of aglycones, glycosides and methyl derivatives [7, 83]. These phytochemicals are found in foods and herbal medicines, and flavonoids and many other phenolic components are effective antioxidants, antibacterial, anticancer, anti-inflammatory, cardioprotective, immune-enhancing, and skin-protective agents against ultraviolet radiation, and are interesting candidates for pharmaceutical and medical applications [82, 84–87]. Over the past few decades, research studies focusing on flavonoids and other phenolic compounds from medicinal plant species have increased dramatically, due to their multiple benefits to human health [83, 88–92]. Most recent reviews have focused on a specific aspect of the effect of flavonoids or phenols on human health.

### Total tannin content (TTC)

**Fig. 4:**
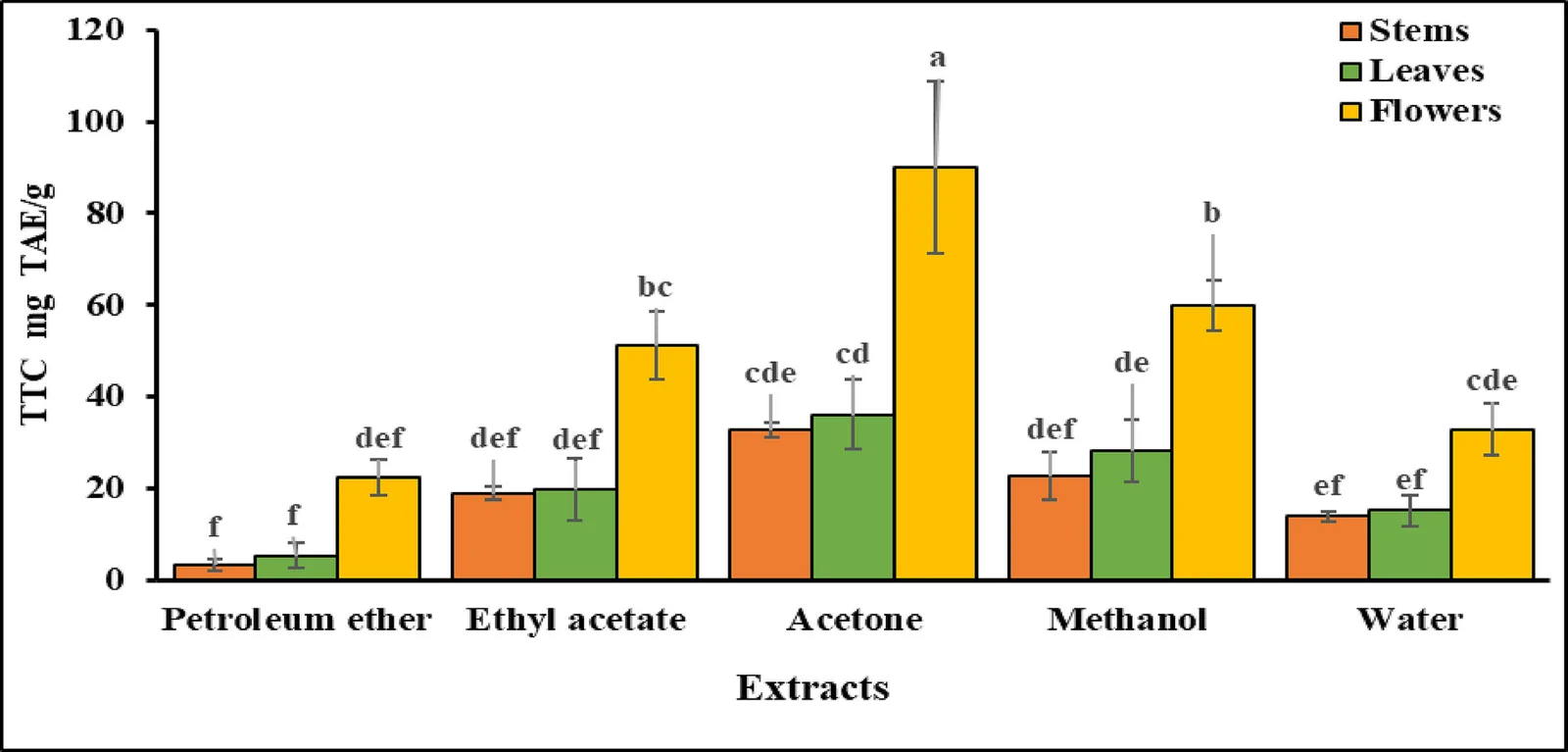
Total tannin content (TTC) is expressed in mg TAE (tannic acid equivalent)/g of extract from different parts of N. oleander, using different solvent fractions in the standard equivalents. Values ​​are the mean ± standard deviation of three replicates. Superscripts: a, b, c, d, e, f (indicate pairwise comparisons to see if they are statistically different)

The results of the present study show that the TTC was higher in the acetone extract (Fig. 4), with the highest TTC in the flowers compared to the leaves and stems. In terms of different parts, the evaluation result also showed that the acetone content in flowers was significantly higher (89.93 ± 18.77 mg TAE/g of extract) than the rest of the solvent fractions (*P* < 0.05), while the petroleum ether extract of stems had the lowest value of 3.45 ± 2.05 mg TAE/g of extract (Table 4). There was no significant difference between the percentage of methanol and the percentage of ethyl acetate in the stem of *N. oleander* (*P* < 0.05) and no significant difference between the percentage of water and the percentage of petroleum ether in the stem and leaves (*P* < 0.05).

Secondary compounds with a wide range of chemical structures, and tannins are abundant in plants and can be categorized into two main types: hydrolyzable tannins and condensed tannins. Due to their capacity for neutralizing free radicals, hydrolyzable and condensed tannins have great promise as biological antioxidants. Tannic acid's antioxidant action is due to its polyphenolic nature, The structure consists of a hydrophobic "core" and a hydrophilic "shell," along with the formation of tannin-protein complexes [93, 94]. Tannin is useful as an antidote for heavy metal and alkaloid poisoning, in addition to its antiviral and antibacterial properties. It can neutralize the body's superoxide free radicals and put off the aging process thanks to its powerful reduced properties [80].

### Antimicrobial activity

The increase in infection morbidity rates worldwide is due to the growing prevalence of infections caused by organisms such as *P. aeruginosa, S. aureus, E. coli,* and *C. albicans* [95, 96]. Among the causes of this increase are antibiotic resistance and a shortage of antimicrobial supplies, especially in poor countries. Thus, during the past few decades, the use of plant extracts to treat microbiological infections has become more and more widespread [97].

The agar well diffusion method was used to analyze the performance of different parts of *N. oleander* extracts (stems, leaves, and flowers) that were extracted using different solvents in suppressing the development of numerous pathogenic microbes such as Gram-positive bacteria (*Staphylococcus aureus* ATCC 6538 and *Bacillus subtilis* ATCC 6633), Gram-negative bacteria (*Pseudomonas aeruginosa* ATCC 9027, *Salmonella Typhimurium* ATCC 14028 and *Escherichia coli* ATCC 11229), and eukaryotic strains such as unicellular fungi (*Candida albicans* ATCC 10231).

**Fig. 5:**
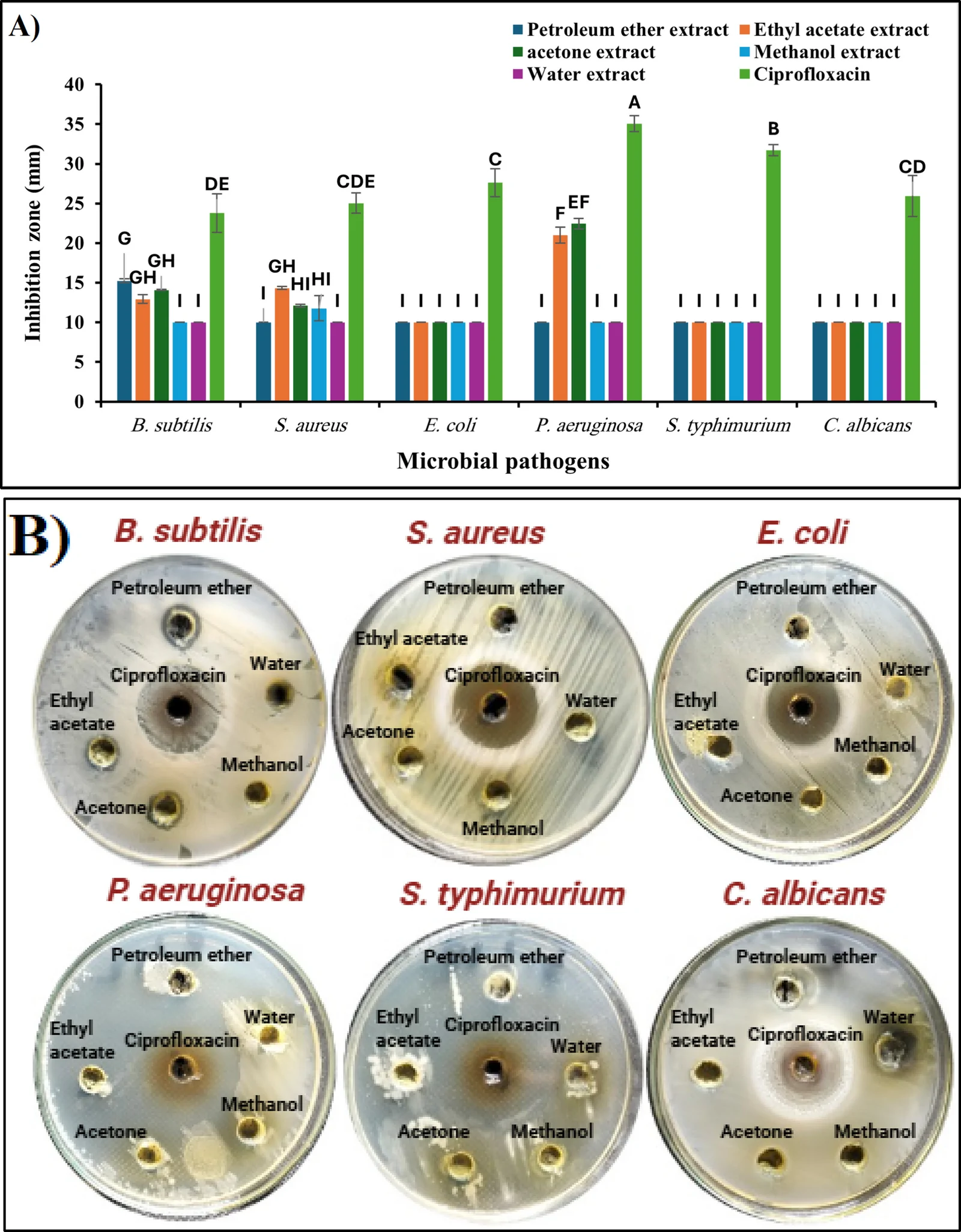
Antimicrobial activity of different solvent extracts of N. oleander stems. **A** Inhibition zones (mm) of the different solvent extracts against the tested microbial pathogens. **B** Representative images of the inhibition zones produced by the different solvent extracts and ciprofloxacin against the tested microbial pathogens. Values are mean ± standard deviation of three replications. Superscript letters Indicate pairwise comparisons whether they are statistically different

The data represented graphically in Fig. 5A, and B summarized the antimicrobial activity of five different solvent extracts of *N. oleander* stems compared with ciprofloxacin as a positive control.

The results shown in Fig. 5A and B demonstrated that the inhibition zones of petroleum ether extract of *N. oleander* stems were 15.23 ± 0.25, 10 ± 0.0, 10 ± 0.0, 10 ± 0.0, 10 ± 0.0 and 10 ± 0.0 mm for *B. subtilis, S. aureus*, *E. coli, P. aeruginosa, S. typhimurium*, and *C. albicans* respectively. On the other hand, the inhibition zones of ethyl acetate extract were 12.9 ± 0.55, 14.3 ± 0.20, 10 ± 0.0, 21 ± 1, 10 ± 0.0 and 10 ± 0.0 mm for *B. subtilis, S. aureus*, *E. coli, P. aeruginosa, S. typhimurium*, and *C. albicans* respectively. Moreover, the inhibition zones of acetone extract were 14.1 ± 0.05, 12.1 ± 017, 10 ± 0.0, 22.4 ± 0.66, 10 ± 0.0 and 10 ± 0.0 mm for *B. subtilis, S. aureus*, *E. coli, P. aeruginosa, S. typhimurium*, and *C. albicans* respectively. Additionally, the effects of methanol extract were 10 ± 0.1, 11.76 ± 1.57, 10 ± 0.0, 10 ± 0.0, 10 ± 0.0 and 10 ± 0.0 mm for *B. subtilis, S. aureus*, *E. coli, P. aeruginosa, S. typhimurium*, and *C. albicans* respectively. While the inhibition zones of water extract were 10 ± 0, 10 ± 0, 10 ± 0, 10 ± 0, 10 ± 0, and 10 ± 0 mm for *B. subtilis, S. aureus*, *E. coli, P. aeruginosa, S. typhimurium*, and *C. albicans* respectively. Furthermore, the effects of ciprofloxacin were 23.76 ± 2.45, 25.03 ± 1.29, 27.6 ± 1.74, 35.03 ± 1.01, 31.7 ± 0.7 and 25.93 ± 2.57 mm for *B. subtilis, S. aureus*, *E. coli, P. aeruginosa, S. typhimurium*, and *C. albicans* respectively.

The antimicrobial activity of *Nerium oleander* stem extracts varied significantly based on the type of solvent used for extraction. The results demonstrated that while some extracts exhibited moderate antibacterial activity, none were as effective as ciprofloxacin, the positive control. The effectiveness of *N. oleander* stem extracts differed depending on the solvent used, with petroleum ether, ethyl acetate, and acetone extracts showing the highest inhibition zones against selected bacterial strains.

In Comparison of solvent extracts, petroleum ether extract exhibited moderate inhibition against *B. subtilis* (15.23 ± 0.25 mm) but was ineffective against all other tested pathogens (*S. aureus, E. coli, P. aeruginosa, S. typhimurium,* and *C. albicans*), where the inhibition zones remained at 10 mm, indicating minimal or no activity. On the other hand, ethyl acetate extract showed better antimicrobial activity, especially against *P. aeruginosa* (21 ± 1 mm), which is a clinically significant pathogen due to its resistance mechanisms. Moderate activity was also observed against *B. subtilis* (12.9 ± 0.55 mm) and *S. aureus* (14.3 ± 0.20 mm). Additionally, acetone extract had comparable effects to ethyl acetate extract, with the highest inhibition against *P. aeruginosa* (22.4 ± 0.66 mm). However, its effects on *B. subtilis* (14.1 ± 0.05 mm) and *S. aureus* (12.1 ± 0.17 mm) were slightly lower. Furthermore, methanol and water extracts demonstrated the least antimicrobial activity, with inhibition zones at or near 10 mm for all tested microorganisms. This suggests that the active antimicrobial compounds in *N. oleander* stems are likely non-polar or semi-polar, making them more extractable in petroleum ether, ethyl acetate, or acetone, rather than in polar solvents like methanol and water.

In Comparison with Ciprofloxacin (Positive Control), Ciprofloxacin exhibited significantly larger inhibition zones across all tested bacteria and *C. albicans*, ranging from 23.76 mm to 35.03 mm. This highlights the limited efficacy of *N. oleander* stem extracts when compared to standard antibiotic treatments. *P. aeruginosa* was the most susceptible to ciprofloxacin (35.03 ± 1.01 mm), whereas the highest inhibition by *N. oleander* extracts (22.4 mm for acetone and 21 mm for ethyl acetate) was still substantially lower. The extracts were particularly weak against *E. coli, S. typhimurium,* and *C. albicans*, with inhibition zones at or near 10 mm, suggesting resistance or minimal effectiveness.

*Nerium oleander* is known to contain several bioactive compounds, such as cardiac glycosides (oleandrin, neriifolin), flavonoids, tannins, and alkaloids, which may contribute to its antimicrobial properties. However, the limited antimicrobial effect observed in this study suggests that the bioactive compounds in the stems may be present in lower concentrations compared to other plant parts like leaves or flowers. These compounds may require specific extraction methods to enhance their antimicrobial potential. The observed antimicrobial activity against *P. aeruginosa* suggests that certain extracts may target Gram-negative bacteria, albeit at lower efficacy than standard antibiotics.

Overall, *N. oleander* stem extracts exhibited limited antimicrobial activity, with the highest effects observed in acetone and ethyl acetate extracts against *P. aeruginosa* and *S. aureus.* However, the extracts were significantly less effective than ciprofloxacin, emphasizing the need for further studies to enhance their antibacterial potency through compound isolation and optimization of extraction methods.

**Fig. 6:**
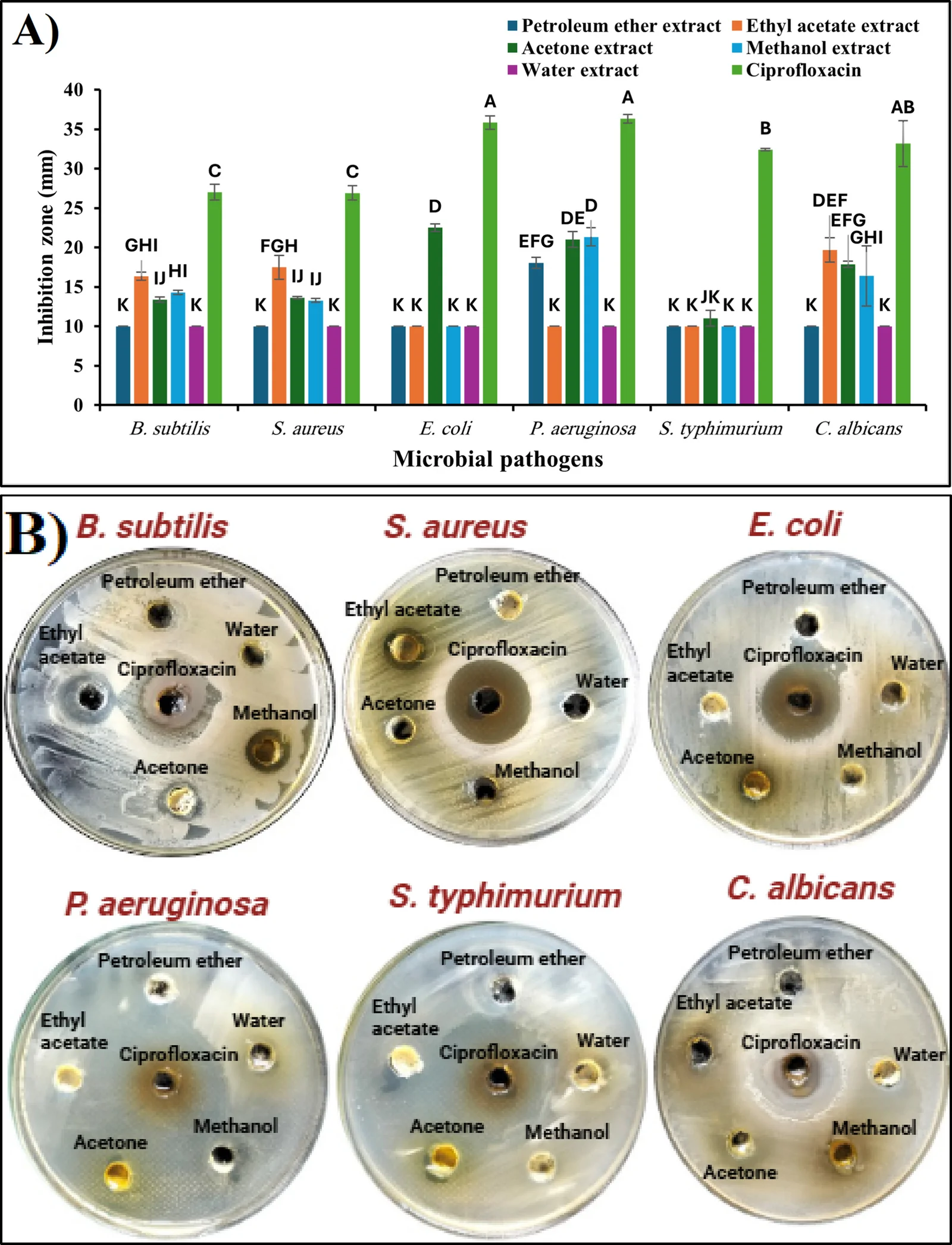
Antimicrobial activity of different solvent extracts of *N. oleander* leaves. **A** Inhibition zones (mm) of the different solvent extracts against the tested microbial pathogens. **B** Representative images of the inhibition zones produced by the different solvent extracts and ciprofloxacin against the tested microbial pathogens. Values are mean ± standard deviation of three replications. Superscript letters Indicate pairwise comparisons whether they are statistically different

The data represented graphically in Fig. 6A and B summarized the antimicrobial activity of five different solvent extracts of *N. oleander* leaves compared with ciprofloxacin as a positive control.

The results shown in Fig. 6A and B demonstrated that the inhibition zones of petroleum ether extract of *N. oleander* leaves were 10 ± 0.0, 10 ± 0.0, 10 ± 0.0, 18.07 ± 0.70, 10 ± 0.0 and 10 ± 0.0 mm for *B. subtilis, S. aureus*, *E. coli, P. aeruginosa, S. typhimurium*, and *C. albicans* respectively. On the other hand, the inhibition zones of ethyl acetate extract were 16.4 ± 0.50, 17.5 ± 1.5, 10 ± 0.0, 10 ± 0.0, 10 ± 0.0 and 19.7 ± 1.5 mm for *B. subtilis, S. aureus*, *E. coli, P. aeruginosa, S. typhimurium*, and *C. albicans* respectively. Moreover, the inhibition zones of acetone extract were 13.4 ± 0.32, 13.6 ± 0.15, 22.5 ± 0.50, 21 ± 1, 11 ± 1 and 17.9 ± 0.41 mm for *B. subtilis, S. aureus*, *E. coli, P. aeruginosa, S. typhimurium*, and *C. albicans* respectively. Additionally, the effects of methanol extract were 14.3 ± 0.26, 13.2 ± 0.25, 10 ± 0.0, 21.3 ± 1.15, 10 ± 0.0 and 16.4 ± 3.81 mm for *B. subtilis, S. aureus*, *E. coli, P. aeruginosa, S. typhimurium*, and *C. albicans* respectively. While the inhibition zones of water extract were 10 ± 0, 10 ± 0, 10 ± 0, 10 ± 0, 10 ± 0 and 10 ± 0 mm for *B. subtilis, S. aureus*, *E. coli, P. aeruginosa, S. typhimurium*, and *C. albicans* respectively. Furthermore, the effects of ciprofloxacin were 27 ± 1, 26.9 ± 0.9, 35.8 ± 0.85, 36.3 ± 0.55, 32.4 ± 0.17 and 33.16 ± 2.92 mm for *B. subtilis, S. aureus*, *E. coli, P. aeruginosa, S. typhimurium*, and *C. albicans* respectively.

The antimicrobial activity of *Nerium oleander* leaf extracts varied depending on the solvent used for extraction, with some extracts demonstrating moderate efficacy against certain pathogens. However, none of the extracts matched the potency of ciprofloxacin, the positive control. The following discussion provides an in-depth comparative analysis of the results.

In Comparison of solvent extracts, petroleum ether extract showed minimal activity against *P. aeruginosa* (18.07 ± 0.70 mm), while no significant inhibition was observed against *B. subtilis, S. aureus, E. coli, S. typhimurium,* or *C. albicans* (all 10 mm). This suggests that non-polar bioactive compounds in *N. oleander* leaves may have limited antibacterial properties. On the other hand, Ethyl Acetate Extract Displayed moderate inhibition against *B. subtilis* (16.4 ± 0.50 mm) and *S. aureus* (17.5 ± 1.5 mm), indicating potential antibacterial compounds targeting Gram-positive bacteria. Its amazing effectiveness against *C. albicans* (19.7 ± 1.5 mm) suggests that it has antifungal characteristics. However, it did not affect *E. coli, P. aeruginosa,* and *S. typhimurium* (10 mm). Additionally, acetone extract showed the highest activity against *E. coli* (22.5 ± 0.50 mm) and *P. aeruginosa* (21 ± 1 mm), demonstrating potential effectiveness against Gram-negative bacteria. It was also moderately effective against *B. subtilis* (13.4 ± 0.32 mm), *S. aureus* (13.6 ± 0.15 mm), *S. typhimurium* (11 ± 1 mm), and *C. albicans* (17.9 ± 0.41 mm). Moreover, Methanol Extract demonstrated activity against *B. subtilis* (14.3 ± 0.26 mm), *S. aureus* (13.2 ± 0.25 mm), *P. aeruginosa* (21.3 ± 1.15 mm), and *C. albicans* (16.4 ± 3.81 mm). However, it was ineffective against *E. coli* and *S. typhimurium* (10 mm). In contrast, water extract showed no antimicrobial activity against any of the tested organisms (all inhibition zones at 10 mm). This suggests that the active antimicrobial compounds in *N. oleander* leaves are non-polar or semi-polar and are not effectively extracted using water.

In Comparison with Ciprofloxacin (Positive Control), Ciprofloxacin exhibited significantly larger inhibition zones against all tested microorganisms, with values ranging from 26.9 mm to 36.3 mm. The highest susceptibility was observed in *P. aeruginosa* (36.3 ± 0.55 mm) and *E. coli* (35.8 ± 0.85 mm), while the lowest was against *S. aureus* (26.9 ± 0.9 mm). The inhibition zones of the most effective *N. oleander* extracts (e.g., acetone extract against *E. coli* at 22.5 mm) were still significantly smaller than those of ciprofloxacin.

In conclusion, the antimicrobial activity of *N. oleander* leaf extracts varied significantly based on the solvent used. Acetone and ethyl acetate extracts showed the highest effectiveness, particularly against *E. coli, P. aeruginosa,* and *C. albicans*.

**Fig. 7:**
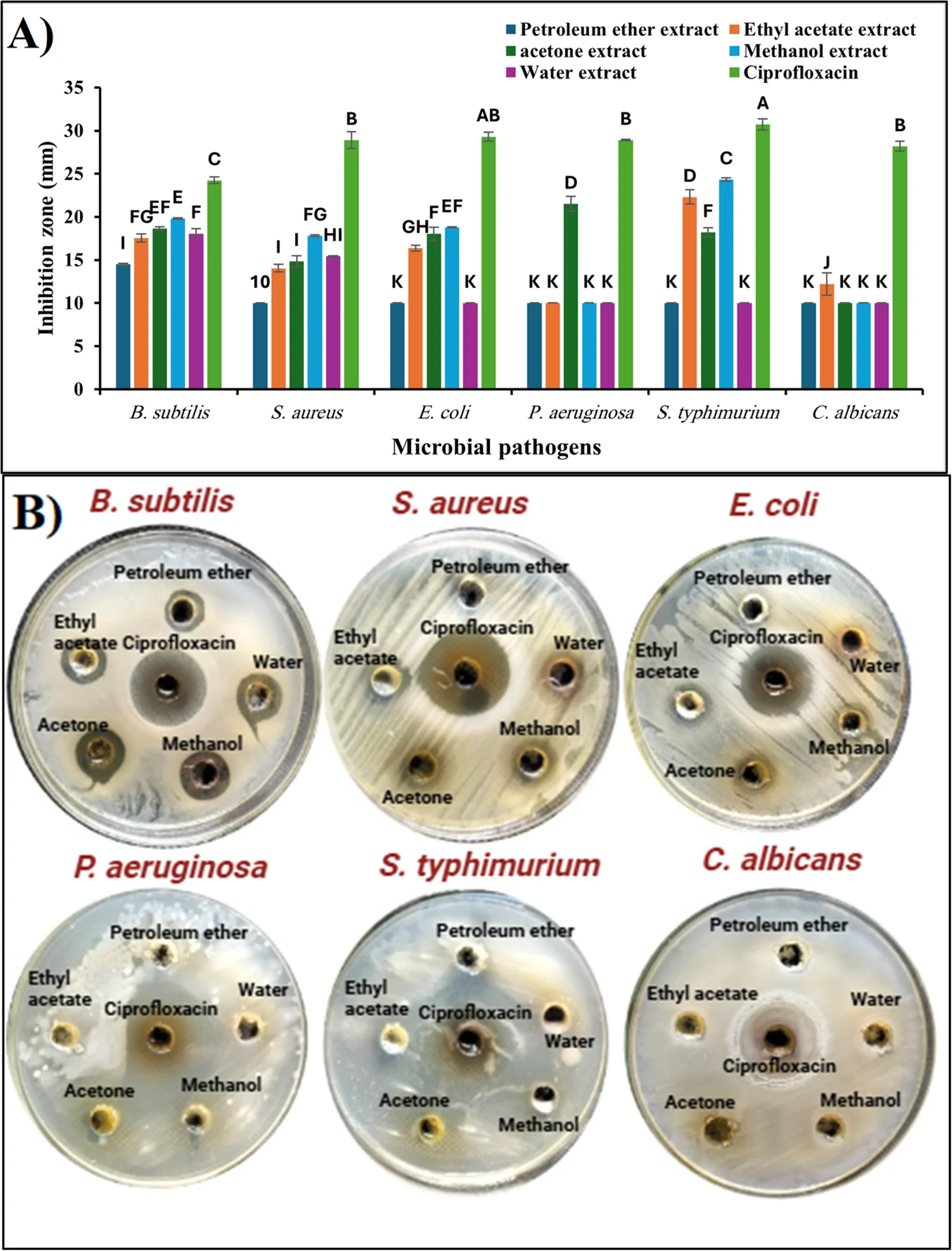
Antimicrobial activity of different solvent extracts of *N. oleander* flowers. **A** Inhibition zones (mm) of the different solvent extracts against the tested microbial pathogens. **B** Representative images of the inhibition zones produced by the different solvent extracts and ciprofloxacin against the tested microbial pathogens. Values are mean ± standard deviation of three replications. Superscript letters Indicate pairwise comparisons whether they are statistically different

The data represented graphically in Fig. 7A and B summarized the antimicrobial activity of five different solvent extracts of *N. oleander* flowers compared with ciprofloxacin as a positive control.

The results shown in Fig. 7A and B demonstrated that the inhibition zones of petroleum ether extract of *N. oleander* flowers were 14.5 ± 0.0, 10 ± 0.0, 10 ± 0.0, 10 ± 0.00, 10 ± 0.0 and 10 ± 0.0 mm for *B. subtilis, S. aureus*, *E. coli, P. aeruginosa, S. typhimurium*, and *C. albicans* respectively. On the other hand, the inhibition zones of ethyl acetate extract were 17.5 ± 0.46, 14 ± 0.45, 16.4 ± 0.32, 10 ± 0.0, 22.3 ± 0.81 and 12.2 ± 1.31 mm for *B. subtilis, S. aureus*, *E. coli, P. aeruginosa, S. typhimurium*, and *C. albicans* respectively. Moreover, the inhibition zones of acetone extract were 18.6 ± 0.23, 14.9 ± 0.63, 18 ± 0.77, 21.5 ± 0.86, 18.2 ± 0.52 and 10 ± 0.0 mm for *B. subtilis, S. aureus*, *E. coli, P. aeruginosa, S. typhimurium*, and *C. albicans* respectively. Additionally, the effects of methanol extract were 19.8 ± 0.1, 17.8 ± 0.1, 18.7 ± 0.05, 10 ± 0, 24.33 ± 0.20 and 10 ± 0 mm for *B. subtilis, S. aureus*, *E. coli, P. aeruginosa, S. typhimurium*, and *C. albicans* respectively. While the inhibition zones of water extract were 18 ± 0.55, 15.4 ± 0.5, 10 ± 0, 10 ± 0, 10 ± 0 and 10 ± 0 mm for *B. subtilis, S. aureus*, *E. coli, P. aeruginosa, S. typhimurium*, and *C. albicans* respectively. Furthermore, the effects of ciprofloxacin were 24.2 ± 0.4, 28.9 ± 0.95, 29.3 ± 0.51, 28.9 ± 0.1, 30.7 ± 0.64 and 28.16 ± 0.56 mm for *B. subtilis, S. aureus*, *E. coli, P. aeruginosa, S. typhimurium*, and *C. albicans* respectively.

The antimicrobial activity of *N. oleander* flower extracts varied based on the solvent used for extraction, with certain extracts exhibiting notable efficacy against selected pathogens. However, none of the extracts demonstrated antimicrobial potency comparable to ciprofloxacin, which served as positive control. The following discussion provides a comparative analysis of these results.

The antimicrobial effects of the *N. oleander* flower extracts differed depending on the extraction solvent petroleum ether extract** s**howed moderate activity against *B. subtilis* (14.5 ± 0.0 mm). No significant inhibition against *S. aureus, E. coli, P. aeruginosa, S. typhimurium,* or *C. albicans* (all at 10 mm). This suggests that non-polar compounds in *N. oleander* flowers may have limited antibacterial properties, mainly affecting *B. subtilis*. On the other hand, ethyl acetate extract demonstrated significant inhibition against *B. subtilis* (17.5 ± 0.46 mm), *S. aureus* (14 ± 0.45 mm), *E. coli* (16.4 ± 0.32 mm), and *S. typhimurium* (22.3 ± 0.81 mm). Also, showed weak inhibition against *C. albicans* (12.2 ± 1.31 mm) and was ineffective against *P. aeruginosa* (10 mm). This suggests the presence of medium-polarity bioactive compounds with antibacterial activity, particularly against Gram-positive bacteria. Furthermore, acetone extract exhibited the broadest spectrum of activity, with inhibition zones ranging from 14.9 ± 0.63 mm (*S. aureus*) to 21.5 ± 0.86 mm (*P. aeruginosa*). Notably effective against *B. subtilis* (18.6 ± 0.23 mm), *E. coli* (18 ± 0.77 mm), and *S. typhimurium* (18.2 ± 0.52 mm). This indicates that acetone effectively extracts bioactive compounds with antibacterial effects, particularly against both Gram-positive and Gram-negative bacteria. Moreover, methanol extracts are the most effective extract overall, with inhibition zones of *B. subtilis* (19.8 ± 0.1 mm), *S. aureus* (17.8 ± 0.1 mm), *E. coli* (18.7 ± 0.05 mm) and *S. typhimurium* (24.33 ± 0.20 mm). While no effect on *P. aeruginosa* or *C. albicans* (10 mm), indicating selectivity in its antimicrobial activity. Additionally, water extract showed moderate inhibition against *B. subtilis* (18 ± 0.55 mm) and *S. aureus* (15.4 ± 0.5 mm). Ineffective against *E. coli, P. aeruginosa, S. typhimurium,* and *C. albicans* (all at 10 mm). Suggests that only a limited number of antimicrobial compounds in *N. oleander* flowers are water-soluble.

In Comparison with Ciprofloxacin (Positive Control), Ciprofloxacin exhibited the highest antimicrobial activity, with inhibition zones ranging from 24.2 mm to 30.7 mm across all tested microorganisms. The highest sensitivity was observed for *S. typhimurium* (30.7 ± 0.64 mm), while *B. subtilis* exhibited the lowest sensitivity (24.2 ± 0.4 mm). Ciprofloxacin was significantly more effective than any *N. oleander* flower extract, suggesting that while *N. oleander* has some antimicrobial properties, but its extracts are not as potent as conventional antibiotics.

In conclusion, among the *N. oleander* flower extracts, the methanol and acetone extracts showed the highest antimicrobial activity, particularly against *B. subtilis, S. aureus, E. coli,* and *S. typhimurium*. The ethyl acetate extract exhibited moderate activity, whereas petroleum ether and water extracts were largely ineffective.

In a comparative Analysis of the Antimicrobial Activity of *N. oleander* stems, leaves, and flowers, Petroleum ether extracts had selective activity, mainly against *B. subtilis* and *P. aeruginosa*. While, ethyl acetate extract, the flower extract had the broadest spectrum of activity, while the leaf extract had significant antifungal properties. On the other hand, Acetone extracts from flowers and leaves exhibited the highest antibacterial activity, while stem extract was selective for *P. aeruginosa*. Additionally, Methanol extracts from flowers were the most effective overall, whereas stem extracts were weak. In contrast, Water extracts were generally inactive, except for flower extracts showing mild antibacterial activity.

In general conclusion of antimicrobial activity, Methanol extracts from flowers are the best overall extract because of their broad-spectrum action. On the other hand, the best defenses against Gram-positive bacteria are floral and leaf ethyl acetate extracts. Acetone extracts of leaves are the most effective against Gram-negative bacteria. Leaf ethyl acetate extract has the strongest antifungal activity. Water extracts from all plant parts are the least effective. *Tordylium maximum* methanol extracts demonstrated inhibitory antibacterial activity against all tested microorganisms, according to Jelena et al. [98], however, water extracts of *T. maximum* did not exhibit inhibitory antimicrobial activity against tested strains. By scavenging free radicals and lowering oxidative stress, flavonoid molecules help prevent several diseases that are caused by an accumulation of free radicals and elevated oxidative stress [99, 100]. Additionally, The phenolic composition, extracted from *N. oleander* which can be explained by adsorption to cell membranes, contact with enzymes, or deprivation of substrate and metal ions, has typically been associated with the inhibitory activity of plant extracts against bacterial infections [101].

**Fig. 8:**
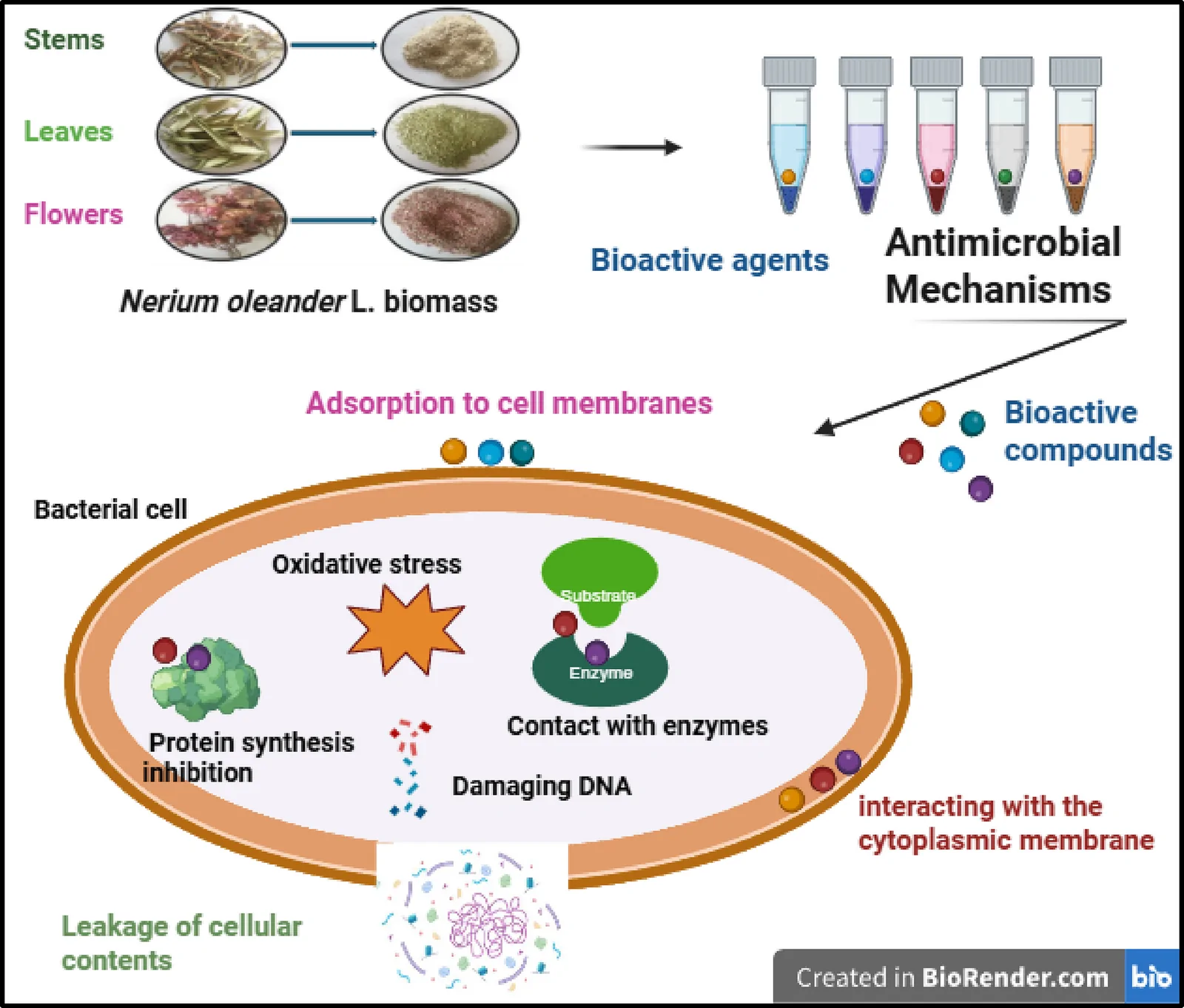
Mechanism of Action of N. oleander extracts against pathogenic bacteria

Medicinal plants such as *N. oleander* contain a wide range of bioactive compounds—including alkaloids, flavonoids, terpenoids, coumarins, tannins, antimicrobial peptides, and steroids—that can serve as alternatives or supplements to traditional antibiotics [102–104]. These compounds exhibit antimicrobial effects through various mechanisms (Fig. 8).

The results of this research showed that different parts of *N. oleander* contain bioactive plant components rich in phenols, tannins, and flavonoids. The bioactive components of northern oleander extracts, and their potential use as natural antioxidants, could be of significant economic value. However, further investigations involving more detailed tests on the extraction, purification, and isolation of biological compounds appear to be needed to identify the components that yield the best biological activities.

## Conclusions

The current study demonstrated that the stems, leaves, and flowers of *Nerium oleander* L. are rich sources of valuable phytochemicals and phytonutrients, supporting their traditional use in ethnomedicine. The different solvents of *N. oleander* contain a significant and appreciable amount of phenols, tannins, and flavonoids conferring to the free radical scavenging activity. Acetone would be the preferred solvent, as it outperforms the others. It is capable of dissolving both hydrophilic and lipophilic compounds, is miscible with water, less volatile, and serves as an effective extractant in bioassays. Various *N. oleander* solvents contain significant amounts of phenols, tannins, and flavonoids, which enhance free radical scavenging activity. Acetone is the optimal solvent, better than other solvents. It can dissolve both hydrophilic and lipophilic compounds, is miscible with water, has low volatility, and serves as an excellent extractant in bioassays. The study proves that different parts of *N. oleander* are rich in carbohydrates, proteins, and lipids, which are not only energy sources but also have good medicinal potential. Moreover, *N. oleander* has health-beneficial nutritional properties, and their addition can improve resistance to diseases. However, further studies are needed on the purification and identification of biochemical compounds for commercial purposes. The methanol extracts from flowers exhibit the most comprehensive antimicrobial activity, making them the most effective overall. Conversely, ethyl acetate extracts from both flowers and leaves provide the strongest defense against Gram-positive bacteria. For Gram-negative bacteria, acetone extracts from leaves demonstrate the highest efficacy. Additionally, the ethyl acetate extract from leaves shows the most potent antifungal activity. Among all extracts, water-based extracts from all plant parts display the weakest antimicrobial effects.

## Data Availability

The data supporting the results of this study can be obtained from the corresponding author upon reasonable request.

## References

[1] Leicach SR, Chludil HD. Plant secondary metabolites: structure–activity relationships in human health prevention and treatment of common diseases. Stud Nat Prod Chem. 2014;42:267–304.

[2] Mahmoud N, Elhaw JE. Biochemical and histopathological studies of the anti-hyperglycemic and anti-hyperlipidemic effects of some extracts of aerial flowering parts of Onopordum alexandrinum Boiss. in alloxan-induced diabetic rats. 2023;66(1):129–142.

[3] Twaij BM, Hasan MNJIJoPB. Bioactive secondary metabolites from plant sources: types, synthesis, and their therapeutic uses. Int J Plant Biol. 2022;13(1):4–14.

[4] Veeresham C. Natural products derived from plants as a source of drugs. J Adv Pharm Technol Res. 2012;3(4):200–1. 10.4103/2231-4040.104709 PMC3560124 23378939

[5] Mahmoud NN, et al. Insight into the biological activities of *Fagonia arabica* L. and its phytochemical constituents. AMB Express. 2025;15(1):114. 10.1186/s13568-025-01918-1 PMC12316655 40751117

[6] Bhutani KK, Gohil VM. Natural products drug discovery research in India: Status and appraisal. Indian J Exp Biol. 2010;48(3):199–207. 21046972

[7] Kumar A, et al. Oleandrin: a cardiac glycosides with potent cytotoxicity. Pharmacognosy Rev. 2013;7(14):131. 10.4103/0973-7847.120512 PMC3841991 24347921

[8] Mahmoud FN, et al. Nutritional assessment, multi-platform phytochemical profiling, and biological activities of Centaurea calcitrapa L. based on GC–MS and HPLC analysis. Sci Rep. 2026;16(1):21614. 10.1038/s41598-026-60984-y PMC13354793 42432029

[9] Mekky AE, et al. Exploring the potential of hydro alcoholic crude extract of beeswax as antibacterial antifungal antiviral antiinflammatory and antioxidant agent. Sci Rep. 2025;15(1):32512. 10.1038/s41598-025-17830-4 PMC12432216 40940367

[10] Shakya AK. Medicinal plants: future source of new drugs. Int J Herb Med. 2016;4(4):59–64.

[11] Abdel Moatamed TSM. The effect of ethanolic extract of Halfa-bar (Cymbopogon Proximus) and parsley (Petroselinum sativum) as an Anti-urolithiasis. Al-Azhar J Agri Res. 2023;48(3):427–40.

[12] Soliman MK, et al. Green synthesis of silver nanoparticles from Bauhinia variegata and their biological applications. Green Processing and Synthesis. 2024;13(1):20240099.

[13] Elkady FM, et al. Unveiling the Launaea nudicaulis (L.) Hook medicinal bioactivities: phytochemical analysis, antibacterial, antibiofilm, and anticancer activities. Front Microbiol. 2024;15:1454623. 10.3389/fmicb.2024.1454623 PMC11484093 39421554

[14] Mekky AE, et al. Antibacterial and antineoplastic MCF-7 and HePG-2 characteristics of the methanolic (80%) clove (Syzygium aromaticum L.) extract. Biomass Conversion and Biorefinery. 2024;14(15):16787–98.

[15] Mahmoud NN, Khader A, Mahmoud E. Green iron oxide nanoparticles and magnetic nanobiochar: enhancing tomato performance, phytochemicals, and root-knot nematode resistance. BMC Plant Biol. 2024;24(1):469. 10.1186/s12870-024-05131-3 PMC11134952 38811862

[16] Mohammed EJ, et al. Biomedical promise of Aspergillus flavus-biosynthesized selenium nanoparticles: a green synthesis approach to antiviral, anticancer, anti-biofilm, and antibacterial applications. Pharmaceuticals. 2024;17(7):915. 10.3390/ph17070915 PMC11279975 39065765

[17] Ghanem SM, et al. Enhancing pan bread quality: nutritional and rheological benefits of partial wheat flour replacement with dahlia tuber powder (Dahlia pinnata L.). Egypt J Chem. 2025;68(5):85–98.

[18] Selim MT, et al. Biological treatment of real textile effluent using Aspergillus flavus and Fusarium oxysporium and their consortium along with the evaluation of their phytotoxicity. J Fungi. 2021;7(3):193. 10.3390/jof7030193 PMC8001397 33803129

[19] Elkhawas I, et al. Study of phytochemical analysis and antimicrobial activity of ethanolic extract of Nigella Sativa L. and Matricaria Chamomilla L. Al-Azhar J Agri Res. 2022;47(2):38–51.

[20] Aldalin HK, et al. Bioactivity screening and molecular identification of Anchusa milleri L. sunflower crud extract for antioxidant, antiviral, antimicrobial, and anticancer properties. Nat Prod Res. 2024;39(23):6780–93. 10.1080/14786419.2024.2390608 39180330

[21] ShElzoghbiy A, et al. Phytochemical analysis of pomegranate peel extract (Ppe) with the evaluation of its efficacy as anti-rancidity of oils. Anti-Bacterial And Anti-Candida Agent. 2022;51(3):1851–66.

[22] Selim MT, et al. Biosynthesis of silver nanoparticles from endophytic fungi and their role in plant disease management. In: Fungal Endophytes Volume II: Applications in Agroecosystems and Plant Protection. Springer; 2025. p. 357–82.

[23] Obaid AN, et al. Green mycosynthesis of a CuO/ZnO heterojunction nanocomposite using Aspergillus terreus and its antibacterial and anti-virulence activity against multidrug-resistant Escherichia coli. Sci Rep. 2026;16(1):12350. 10.1038/s41598-026-44775-z PMC13079814 41974827

[24] Barbosa R, Fontenele-Neto J, Soto-Blanco BJRivs. Toxicity in goats caused by oleander (*Nerium oleander*). Res Vet Sci. 2008;85(2):279–81. 10.1016/j.rvsc.2007.10.004 18031775

[25] Pandey A, et al. Phytochemical and pharmacological attributes of Nerium oleander: a review. Curr Nutr Food Sci. 2024;20(5):570–85.

[26] Derwich E, et al. Antibacterial activity and chemical composition of the essential oil from flowers of Nerium oleander. Electronic J Environ Agri Food Chem. 2010;9(6):1074.

[27] Hussain, M. and M. Gorsi, Antimicrobial activity of Nerium oleander Linn. 2004.

[28] Govind P, Satish N, Shobhit S. Antiulcer activity of methanolic leaves extract of Nerium indicum Mill. Int J Biomed Res. 2010;1:55–61.

[29] Singhal KG, Gupta GDJAPjotm. Hepatoprotective and antioxidant activity of methanolic extract of flowers of *Nerium oleander* against CCl4–induced liver injury in rats. Asian Pac J Trop Med. 2012;5(9):677–85. 10.1016/S1995-7645(12)60106-0 22805717

[30] Calderón-Montaño JM, et al. A hydroalcoholic extract from the leaves of Nerium oleander inhibits glycolysis and induces selective killing of lung cancer cells. Planta Med. 2013;79(12):1017–23. 10.1055/s-0032-1328715 23824549

[31] Ayouaz S, et al. LC-DAD-ESI-MS/MS analysis and cytotoxic and antiproliferative effects of chlorogenic acid derivative rich extract from *Nerium oleander* L. pink flowers. Food Funct. 2021;12(8):3624–34. 10.1039/d0fo02640a 33900304

[32] Hassan M, et al. Studies on the antidiarrhoeal, antimicrobial and cytotoxic activities of ethanol-extracted leaves of yellow oleander (*Thevetia peruviana*). Open Vet J. 2011;1:28–31. PMC4655751 26623276

[33] Sakthivadivel M, et al. Larvicidal activity of Wrightia tinctoria R. BR.(Apocynaceae) fruit and leaf extracts against the filarial vector Culex quinquefasciatus Say (Diptera: Culicidae). Asian Pac J Trop Dis. 2014;4:S373–7.

[34] Bandara V, et al. A review of the natural history, toxinology, diagnosis and clinical management of Nerium oleander (common oleander) and Thevetia peruviana (yellow oleander) poisoning. Toxicon. 2010;56(3):273–81. 10.1016/j.toxicon.2010.03.026 20438743

[35] Dhawan B, Rastogi R. Recent developments from Indian medicinal plants. In: The medicinal plant industry. Routledge; 2017. p. 185–208.

[36] Tahir K, et al. Nerium oleander leaves extract mediated synthesis of gold nanoparticles and its antioxidant activity. Mater Lett. 2015;156:198–201.

[37] Sastry KS, et al. Nerium oleander (Oleander). In: Encyclopedia of Plant Viruses and Viroids. Springer; 2020. p. 1603–4.

[38] Jabli M, et al. Physicochemical characteristics and dyeing properties of lignin-cellulosic fibers derived from Nerium oleander. J Mol Liquids. 2018;249:1138–44.

[39] Kars MD, et al. Exploring a natural MDR reversal agent: potential of medicinal food supplement Nerium oleander leaf distillate. Asian Pac J Trop Biomed. 2013;3(8):644–9. 10.1016/S2221-1691(13)60130-6 PMC3703559 23905023

[40] del Carmen Juárez-Vázquez M, et al. Ethnobotany of medicinal plants used in Xalpatlahuac. Guerrero, México. 2013;148(2):521–7. 10.1016/j.jep.2013.04.048 23665055

[41] Mekky AE, et al. Phytochemical analysis of Centaurea calcitrapa L Aerial flowering parts serial solvent extracts and its antibacterial and antioxidant activities. Life. 2024;14(7):900. 10.3390/life14070900 PMC11278314 39063653

[42] Pizzale L, et al. Antioxidant activity of sage (Salvia officinalis and S fruticosa) and oregano (Origanum onites and O indercedens) extracts related to their phenolic compound content. J Sci Food Agri. 2002;82(14):1645–51.

[43] Arunachalam K, Parimelazhagan T. Evaluation of nutritional composition and antioxidant properties of underutilized Ficus talboti King fruit for nutraceuticals and food supplements. J Food Sci Technol. 2014;51:1260–8. 10.1007/s13197-012-0647-6 PMC4062692 24966418

[44] Premnath D, Gomez P. Antifungal and anti bacterial activities of chemical constituents from Heliotropium indicum Linn. Plant. Drug Invention Today. 2012;4(11):564.

[45] Arunachalam K, et al. Nutritional analysis and antioxidant activity of Palmyrah (Borassus flabellifer L.) seed embryo for potential use as food source. Food Sci Biotechnol. 2011;20:143–9.

[46] Krishna CS, et al. Evaluation of nutraceutical properties of Laportea interrupta (L.) Chew. Food Sci Biotechnol. 2014;23:577–85.

[47] Indrayan, A., et al., Determination of nutritive value and analysis of mineral elements for some medicinally valued plants from Uttaranchal. Curr Sci. 2005:1252–1255.

[48] Herborne JB. Phytochemical Methods. A Guid to Mod Tech Plant Anal. 1973;2:5–11.

[49] Chandra S, et al. Assessment of total phenolic and flavonoid content, antioxidant properties, and yield of aeroponically and conventionally grown leafy vegetables and fruit crops: A comparative study. Evidence-Based Complementary and Alternative Medicine. 2014;2014(1):253875. 10.1155/2014/253875 PMC3980857 24782905

[50] Aryal S, et al. Total phenolic content, flavonoid content and antioxidant potential of wild vegetables from Western Nepal. Plants. 2019;8(4):96. 10.3390/plants8040096 PMC6524357 30978964

[51] Makkar HP. Quantification of tannins in tree and shrub foliage: a laboratory manual. Springer Science & Business Media; 2013.

[52] Humphries RM, et al. CLSI methods development and standardization working group best practices for evaluation of antimicrobial susceptibility tests. J Clin Microbiol. 2018;56(4):10–128. 10.1128/jcm.01934-17. PMC5869819 29367292

[53] Selim MT, et al. Statistical optimization of the fermentation factors for increased biorefinery of corn-steep water effluent to lactic acid by Enterococcus faecium strain WH51–1. Biomass Conversion and Biorefinery. 2022;14(21):27829–52.

[54] Selim MT, et al. Nutrient-free biorefinery of corn steep water into lactic acid by *Bacillus licheniformis* OP16-2 under thermo-alkaline conditions with a pilot-scale assessment. Sci Rep. 2026;16(1):4357. 10.1038/s41598-026-35828-4 PMC12864893 41629402

[55] Azmir J, et al. Techniques for extraction of bioactive compounds from plant materials: a review. J Food Eng. 2013;117(4):426–36.

[56] Saeed N, et al. Antioxidant activity, total phenolic and total flavonoid contents of whole plant extracts Torilis leptophylla L. BMC Complement Alternative Med. 2012;12:1–12. 10.1186/1472-6882-12-221 PMC3524761 23153304

[57] Ammar I, et al. Phenolic content and antioxidant activity of cactus (*Opuntia ficus-indica* L.) flowers are modified according to the extraction method. Ind Crops Prod. 2015;64:97–104.

[58] Zaid R, et al. Phytochemical analyses and toxicity of *Nerium oleander* (Apocynaceae) leaf extracts against *Chaitophorus leucomelas* Koch, 1854 (Homoptera: Aphididae). J Saudi Soc Agric Sci. 1854;21(5):310–7.

[59] Kalia K, et al. Effects of extraction methods on phenolic contents and antioxidant activity in aerial parts of *Potentilla atrosanguinea* Lodd. and quantification of its phenolic constituents by RP-HPLC. J Agric Food Chem. 2008;56(21):10129–34. 10.1021/jf802188b 18841977

[60] Hayouni EA, et al. The effects of solvents and extraction method on the phenolic contents and biological activities in vitro of Tunisian *Quercus coccifera* L. and *Juniperus phoenicea* L. fruit extracts. Food Chem. 2007;105(3):1126–34.

[61] Nandhakumar E, Indumathi P. In vitro antioxidant activities of methanol and aqueous extract of Annona squamosa (L.) fruit pulp. Innov Acupunct Med. 2013;6(3):142–8. 10.1016/j.jams.2012.09.002 23787283

[62] Jones WP, Kinghorn AD. Extraction of plant secondary metabolites. 2005:323–351. 10.1007/978-1-61779-624-1_13 22367903

[63] Adel KM et al. Applied study on the development of healthy yoghurt drinks using stevia sugar and strawberry pulp. J Food Sci Technol. 2026:1–12.

[64] Quispe-Condori S, et al. Obtaining β-caryophyllene from Cordia verbenacea de Candolle by supercritical fluid extraction. 2008;46(1):27–32.

[65] Ishida BK, Ma J, Chan BJPa. A simple, rapid method for HPLC analysis of lycopene isomers. Phytochem Anal. 2001;12(3):194–8. 10.1002/pca.576 11705025

[66] Kaleta A, Górnicki K, Criteria of determination of safe grain storage time. 2013:295.

[67] Imran M, et al. Analysis of nutritional components of some wild edible plants. J Chem Soc Pakistan. 2007;29(5):500.

[68] Latique, S., et al. The effect of foliar application of Ulva rigida extract on the growth and biochemical parameters of wheat plants. in E3S Web of Conferences. 2021. EDP Sciences.

[69] Fagbohun E, O Lawal, M Ore. The proximate, mineral and phytochemical analysis of the leaves of Ocimum gratissimum L., Melanthera scandens A. and Leea guineensis L. and their medicinal value. 2012.

[70] Kadam MPV, et al. Phytopharmacopoeial specifications of Garcinia indica fruit rinds. Pharmacognosy J. 2012;4(31):23–8.

[71] Larcher WJApp. Physiological plant ecology, vol. 18. 1996. p. 183–4.

[72] Smith YA. Determination of chemical composition of Senna-siamea (Cassia leaves). 2009;8(2):119–121.

[73] Charalambous G. Shelf life studies of foods and beverages: chemical, biological, physical, and nutritional aspects. 1993.

[74] Vaughan JG, Judd PA. The Oxford book of health foods. Oxford University Press; 2003.

[75] Escudero NL, et al. Taraxacum officinale as a food source. 2003;58:1–10.

[76] Redha AA. Phytochemical investigations of Nerium oleander L. leaves and flowers. Int J Sci Res Chem Sci. 2020;7(4):1–4.

[77] Fraga CG, et al. The effects of polyphenols and other bioactives on human health. 2019;10(2):514–28. 10.1039/c8fo01997e 30746536

[78] de Lima Cherubim DJ, et al. Polyphenols as natural antioxidants in cosmetics applications. 2020;19(1):33–7. 10.1111/jocd.13093 31389656

[79] Wen K, et al. Recent research on flavonoids and their biomedical applications. 2021;28(5):1042–66. 10.2174/0929867327666200713184138 32660393

[80] Tong Z, et al. Biological function of plant tannin and its application in animal health. 2022;8:803657. 10.3389/fvets.2021.803657 PMC8784788 35083309

[81] Bakir Çilesizoğlu N, et al. Qualitative and quantitative phytochemical screening of Nerium oleander L. extracts associated with toxicity profile. Sci Rep. 2022;12(1):21421. 10.1038/s41598-022-26087-0 PMC9742154 36504046

[82] Kumar S, Pandey AK. Chemistry and biological activities of flavonoids: an overview. Sci World J. 2013;2013(1):162750. 10.1155/2013/162750 PMC3891543 24470791

[83] Ahmed SI, et al. Pharmacologically active flavonoids from the anticancer, antioxidant and antimicrobial extracts of Cassia angustifolia Vahl. BMC Complement Alternative Med. 2016;16:1–9. 10.1186/s12906-016-1443-z PMC5106795 27835979

[84] Chen X, Dang T-TT, Facchini PJJP. Noscapine comes of age. 2015;111:7–13. 10.1016/j.phytochem.2014.09.008 25583437

[85] Działo M, et al. The potential of plant phenolics in prevention and therapy of skin disorders. 2016;17(2):160. 10.3390/ijms17020160 PMC4783894 26901191

[86] Andreu L, et al. Antioxidant properties and chemical characterization of Spanish Opuntia ficus-indica Mill. cladodes and fruits. J Sci Food Agri. 2018;98(4):1566–73. 10.1002/jsfa.8628 28833143

[87] Meng X-H, et al. Antioxidative flavan-3-ol dimers from the leaves of *Camellia fangchengensis*. J Agric Food Chem. 2018;66(1):247–54. 10.1021/acs.jafc.7b04572 29232949

[88] Zhishen J, Mengcheng T, Jianming WJFc. The determination of flavonoid contents in mulberry and their scavenging effects on superoxide radicals. Food Chem. 1999;64(4):555–9.

[89] Oki T, et al. Involvement of anthocyanins and other phenolic compounds in radical-scavenging activity of purple-fleshed sweet potato cultivars. 2002;67(5):1752–6.

[90] Okpuzor, J., et al., In vitro investigation of antioxidant phenolic compounds in extracts of Senna alata. 2009.

[91] Wink MJM. Modes of action of herbal medicines and plant secondary metabolites. 2015;2(3):251–86. 10.3390/medicines2030251 PMC5456217 28930211

[92] Wang J, et al. Variations in chemical fingerprints and major flavonoid contents from the leaves of thirty-one accessions of Hibiscus sabdariffa L. 2016;30(6):880–7. 10.1002/bmc.3623 PMC5061090 26394363

[93] Onyekere PF, et al. Biological roles of phytochemicals. In: Phytochemistry. Apple Academic Press; 2018. p. 119–52.

[94] Parmar F, et al. In vitro antioxidant and anticancer activity of Mimosa pudica linn extract and L-Mimosine on lymphoma daudi cells. 2015;1:100–4.

[95] Rakelly de Oliveira D, et al. In vitro antimicrobial and modulatory activity of the natural products silymarin and silibinin. BioMed Res Int. 2015;2015(1):292797. 10.1155/2015/292797. PMC4377387 25866771

[96] Shaker A, et al. Polyurethane nanofibrous scaffolds for wound repair: from electrospinning fabrication to biological performance evaluation. BMC Biotechnol. 2026;26(1):43. 10.1186/s12896-026-01114-4 PMC13063895 41814283

[97] Bibi Y, et al. Antibacterial activity of some selected medicinal plants of Pakistan. 2011;11:1–7. 10.1186/1472-6882-11-52 PMC3141602 21718504

[98] MatejiÄ JS, et al. Total phenolic and flavonoid content, antioxidant and antimicrobial activity of extracts from Tordylium maximum. J Appl Pharm Sci. 2013;3(1):055–9.

[99] El Rabey HA, Almutairi FM. The antioxidant, antidiabetic, antimicrobial and anticancer constituents of Artemisia species. Nat Prod Res. 2025;39(6):1685–95. 10.1080/14786419.2024.2384082 39056203

[100] Mahmoud NN, Selim MT. Phytochemical analysis and antimicrobial activity of *Silybum marianum* L. via multi-solvent extraction. AMB Express. 2025;15(1):122. 10.1186/s13568-025-01925-2 PMC12367635 40833703

[101] El Mannoubi I. Impact of different solvents on extraction yield, phenolic composition, in vitro antioxidant and antibacterial activities of deseeded Opuntia stricta fruit. J Umm Al-Qura University Appl Sci. 2023;9(2):176–84.

[102] AlSheikh HMA, et al. Plant-based phytochemicals as possible alternative to antibiotics in combating bacterial drug resistance. 2020;9(8):480. 10.3390/antibiotics9080480 PMC7460449 32759771

[103] Fazly Bazzaz BS, et al. Human antimicrobial peptides: Spectrum, mode of action and resistance mechanisms. Int J Peptide Res Ther. 2021;27:801–16.

[104] Khameneh B, et al. Phytochemicals: a promising weapon in the arsenal against antibiotic-resistant bacteria. Antibiotics (Basel). 2021. 10.3390/antibiotics10091044. PMC8472480 34572626

